# Shift-adjusted Neyman–Pearson classifiers via single index modeling (SACSIM)

**DOI:** 10.1093/biomtc/ujag138

**Published:** 2026-08-10

**Authors:** Jiaming Qiu, Ying-Qi Zhao, Yingye Zheng

**Affiliations:** Biostatistics Program, Public Health Sciences Division, Fred Hutchinson Cancer Center, Seattle, WA 98109, United States; Biostatistics Program, Public Health Sciences Division, Fred Hutchinson Cancer Center, Seattle, WA 98109, United States; Biostatistics Program, Public Health Sciences Division, Fred Hutchinson Cancer Center, Seattle, WA 98109, United States

**Keywords:** data shift, density ratio, Neyman–Pearson classifier, sample weighting, unlabeled target data

## Abstract

Neyman–Pearson (NP) classifiers, which aim to maximize the clinical benefit while adhering to risk constraints, are crucial in many practical fields, including early cancer detection. However, applying these classifiers can be challenging due to discrepancies between the data distributions of the source and target populations. The potential impact can be disproportionately severe for under-represented groups. We propose a semi-parametric model-based approach for adapting NP classifier decision rules to different populations while equitably controlling classification errors specific to clinical applications. Our method involves a shift-adjustment strategy that leverages a small unlabeled sample from the target population, along with minimal auxiliary information and the labeled source data. This approach enhances the applicability of the learned decision rules and ensures they are consistently tailored for the target population. We demonstrate the performance through theoretical studies and simulations and illustrate the approach with an example of a prostate cancer study.

## 1 Introduction

In early cancer detection or surveillance, a biomarker-based test may fail to detect a malignant condition or its progression, or may prompt a healthy patient to undergo invasive and costly follow-up testing. The two error types often carry markedly different clinical consequences. The Neyman–Pearson (NP) classifiers are particularly well suited to such medical decision problems, controlling one type of classification error [such as the false positive rate (FPR)] below a prespecified level while maximizing detection power [such as the true positive rate (TPR)] (Cannon et al., 2002; Scott and Nowak, 2005). For example, in prostate cancer detection, NP classifiers are sought to minimize unnecessary referrals for invasive biopsy while maintaining a high detection rate of clinically significant disease (Sanda et al., 2017; Wang et al., 2020).

A woefully neglected step in developing medical decision rules concerns their generalizability in the presence of distributional data discrepancy across populations, a phenomenon broadly referred to as *data shift* (e.g., Moreno-Torres et al., 2012). Differences in demographic composition, clinical practice, and study design induce changes in the marginal distribution of covariates (X-shift), while biological heterogeneity and unmeasured confounders may alter the conditional relationship between covariates and outcomes ($Y \mid X$-shift). For example, Black men have a higher overall incidence of prostate cancer than men of European ancestry (Hinata and Fujisawa, 2022). Some tumor molecular features, such as PTEN loss and ERG expression, may also differ across populations (Tosoian et al., 2017). At a given screening marker level, disease risk may differ across populations (Lee et al., 2024). These shifts can degrade model performance and, for NP classifiers, jeopardize the desired control of clinically important error rates in the target population. Therefore, when an NP classifier is transported across populations, its clinically relevant error rate must still be controlled in the target population, which generally cannot be guaranteed if source–target differences are ignored.

Since labeled data from the *target population* are often limited, recalibrating based on the richer data from the *source population* used for deriving the rule is often necessary. This motivates procedures that borrow strength from the source domain while correcting for data shifts, so that the desired error control is preserved after transport.

A standard approach to handling such distributional discrepancies is to reweight source observations using the target-to-source density ratio. Methods for estimating this ratio are well established under *X*-shift. These include direct density ratio estimation via divergence minimization (Kanamori et al., 2009; Sugiyama et al., 2008; Yamada et al., 2013) and approaches based on modeling selection probabilities (Bickel et al., 2007; Zadrozny, 2004). By construction, these methods target the ratio of density functions for *X* in the two populations and implicitly assume that the conditional distribution $(Y \mid X)$ is invariant across populations (Kouw and Loog, 2021).

A complementary line of work, rooted in the empirical likelihood framework (Owen, 1990), incorporates external summary statistics to recalibrate risk models under population differences. Recent contributions use such constraints to adjust absolute risk estimates (Chu et al., 2023; Han and Lawless, 2019; Zheng et al., 2021) and extend to heterogeneous populations (Cheng et al., 2023; Huang et al., 2023; Sheng et al., 2022). While these methods partially address shifts in outcome prevalence or in aspects of $(Y \mid X)$, they typically rely on low-dimensional summary constraints on *X*, which may not capture complex distributional differences in the covariates. Moreover, they do not explicitly construct weights that jointly account for both covariate and conditional risk shifts.

In many practical settings, however, both types of shift arise simultaneously. As a result, deriving a transportable NP classifier requires accounting for changes in the joint distribution of $(X, Y)$. A unified framework that integrates both remains underdeveloped, particularly for NP classification, where valid error control depends on the target joint distribution.

To address the aforementioned challenges, we propose the shift-adjusted Neyman–Pearson classifier via single index modeling (SACSIM), a sample-weighting approach for deriving an adjusted NP classifier-based decision rule. We first factorize the joint density ratio into the density ratio of covariates and that of the conditional risk $Y|X$. Hence, the sampling weights explicitly account for both types of data shifts, thereby enhancing the applicability of derived rules. Specifically, we follow the density ratio framework to address *X*-Shifts by leveraging the unlabeled data from both the source and target populations to estimate covariate distributions. To robustly model the conditional risk $Y|X$ and account for the $Y|X$-shift, we employ a semi-parametric model for the risk $Y|X$ that incorporates potential $Y|X$-shift, combining a single index model with a local smoothing technique within the class of plug-in classifiers. It alleviates the dependence on stringent modeling assumptions while maintaining tractability and interpretability. The proposed risk model is estimated using labeled samples from the source population along with the marginal prevalence $\mathbb {P}(Y=1)$ known for the target population. The density ratio for the conditional $Y|X$ can then be derived based on the model. An NP-classifier for the target population can then be derived by combining both types of weights to fully address the data shift. For inference, we extend the theoretical treatment of generic plug-in NP classifiers (e.g., Scott and Nowak, 2005; Tong, 2013) to the data shift situation. Under mild assumptions, we show the proposal consistently controls the specific classification error, such as the FPR, while maximizing the TPR for the target population based on unlabeled samples.

This paper is structured as follows: Section 2 introduces the problem and details the proposed method, with Subsection 2.2 discussing estimators for the plug-in rules, while Section 3 provides a theoretical treatment for the consistency. Sections 4 and 5 showcase the performance in simulated and real-world examples, respectively.

## 2 NP classifier under data shift

Let $X \in \mathcal {X}\subset \mathbb {R}^d$ and $Y \in \left\lbrace 0, 1 \right\rbrace$ be the risk factors and the outcome indicator, respectively. We consider a binary decision rule/classifier $R: \mathcal {X}\rightarrow \left\lbrace 0, 1 \right\rbrace$ predicting *Y* based on *X*. An NP rule aims to maximize the gain while controlling risk with a minimally acceptable limit. In our clinical setup, the objective function can be characterized by TPR and FPR, respectively, defined as (abbrev. in upright Roman font) $\mathrm{TPR}(R) {:=}\mathbb {E}\left(R(X)| Y = 1\right)$ and $\mathrm{FPR}(R) {:=}\mathbb {E}\left(R(X)| Y = 0\right)$. We aim to find an optimal algorithm to rule in high-risk patients for treatments that


(1) \begin{eqnarray*}
\text{maximize } \mathrm{TPR}(R)\text{ subject to } \mathrm{FPR}(R) \le \alpha ,
\end{eqnarray*}

or to rule out low-risk patients to avoid unnecessary procedures that


(2) \begin{eqnarray*}
\text{minimize } \mathrm{FPR}(R) \text{ subject to }\mathrm{TPR}(R) \ge 1 - \beta ,
\end{eqnarray*}

for some level of control $0 < \alpha < 1$ and $0 < \beta < 1$. We focus our discussion on (1) because (1) and (2) are equivalent up to flipping the outcome label: $\mathbb {E}(R(X)| Y = y) = 1 - \mathbb {E}(1 - R(X)| 1 - Y = 1 - y)$ for $y = 0, 1$.

Problem (1) is closely tied to the most powerful test subject to a type I error rate no greater than $\alpha$. Therefore, the NP lemma indicates that the optimal rule is the likelihood ratio rule (see Subsection S3.1 of the Supplement). Because the likelihood ratio is a monotone function of posterior risk, the optimal rule $R^{*}$ takes the following form:


(3) \begin{eqnarray*}
R^{*}(X) &=& 1 \text{ if } \mathbb {P}\left(Y = 1|X\right) > c; \\R^{*}(X) &=& 0 \text{ if } \mathbb {P}\left(Y = 1|X\right) < c;
\end{eqnarray*}

for some constant *c* such that $\mathbb {E}\left(R^{*}(X)| Y = 0\right) = \alpha$.

As demonstrated in Section S1 of the Supplement, the NP classifier (3) is sensitive to data shift because it relies on correctly specifying both the conditional risk $Y|X$ and the cutoff threshold *c*. The classifier would fail when $Y|X$ shifts, leading to a misspecified risk for the target population. It also fails when *X* shifts so that the cutoff cannot be properly specified.

### 2.1 Semi-parametric model-based shift adjustment by sample weighting

Our analytical goal is to provide a framework for deriving an NP classifier that achieves optimized performance subject to equal risk control in the presence of *X*- and $Y|X$-shifts. We propose a sample weighting shift-adjustment method based on semi-parametric risk and selection probability models. Let a selection variable $S \in \left\lbrace 0, 1 \right\rbrace$ indicate whether the observation was drawn from the source (e.g., cohort $S =1$) or the target population ($S = 0$). The shift-adjustment problem is formulated as solving (1) under $X, Y|S = 0$ given jointly $X, Y| S = 1$ and marginally $X|S = 0$ without $Y|S = 0$.

Covariate shift is relatively easy to tackle utilizing the ratio of densities. Suppose both $f_{X, \mathrm{sr}}, f_{X, \mathrm{tg}}$ (the density function of $X|S = 1$ and $X|S = 0$, respectively) are bounded away from 0 on $\mathcal {X}$, we can write $w_X= f_{X, \mathrm{tg}}/ f_{X, \mathrm{sr}}: \mathcal {X}\rightarrow \mathbb {R}^+$, which provides $\mathbb {E}[g(X)| S = 0] = \mathbb {E}[w_X\left(X\right) g(X)| S = 1]$ for any measurable function *g*. Thus, if there is only *X*-shift, it suffices to weight the sample using $w_X$ and proceed as usual. The $w_X$ is obtained by Bayes’ rule, $w_X\left(x\right) = ((1 - \psi ) \mathbb {P}\left(S = 1\right))/(\psi \mathbb {P}\left(S = 0\right))$, where


(4) \begin{eqnarray*}
\psi = \psi \left(x\right):= \mathbb {P}\left(S = 1| X = x\right)
\end{eqnarray*}

is the *propensity model* (see, e.g., Bickel et al., 2009; Cranmer et al., 2016) that tracks the probability of selection based on covariates. The NP classifier obtained from the weighted sample will be consistent for the target population.

To address $Y|X$-shift, we further find some $w_{Y|X}$ such that $\mathbb {E}[g(X, Y)| S = 0] = \mathbb {E}[w_{Y|X}\left(X, Y\right) w_X\left(X\right) g(X, Y)| S = 1]$ for all measurable functions *g*. Notice that *Y* is binary, we can set $w_{Y|X}(x, y) {:=}\mathbb {P}(Y = y| X = x, S = 0) / \mathbb {P}(Y = y| X = x, S = 1)$ for $x \in \mathcal {X}$ and $y = 0, 1$. In combination, we weight the sample by the product of the *X*-ratio and $Y| X$-ratio, which is effectively $\mathbb {P}\left(X, Y| S=0\right) / \mathbb {P}\left(X, Y|S = 1\right) \propto \mathbb {P}\left(S = 1| X, Y\right) /\mathbb {P}\left(S = 0| X, Y\right)$, i.e., the *selection odds* of source to target. Subsequently, to obtain a likelihood-based rule with an FPR of $\alpha$ for the target population, it suffices to find *c*, such that


(5) \begin{eqnarray*}
&& \alpha \mathbb {P}\left(Y = 0| S = 0\right)\\&&\quad = \mathbb {E}[\mathbb {1}_{\left\lbrace \eta (X) \ge c \right\rbrace } (1 - Y) w_{Y|X}\left(X, Y\right)w_X\left(X\right)| S = 1],
\end{eqnarray*}

where $\eta (x) = \mathbb {P}\left(Y = 1| X = x, S = 0\right)$ is the risk for the target population. Note that the RHS of (5) is a function of *c* decreasing from $\mathbb {P}\left(Y = 0| S = 0\right)$ to 0. A solution $c^{*}$ to (5) provides a shift-adjusted optimal rule $R^{*}(x) = \mathbb {1}_{\left\lbrace \eta (x) \ge c^{*} \right\rbrace }$ for the target population.

If no additional information is available, $w_{Y|X}$ is intractable without labeled target-population data. We therefore assume a common single index structure across populations, with any $Y|X$ difference represented through a baseline shift driven by factors not included in the model. That is,


(6) \begin{eqnarray*}
\mathbb {P}\left(Y = 1| X, S\right) = \varphi \left(\Delta _0(1 - S) + {\theta }_{0}^{T}X\right),
\end{eqnarray*}

where $\varphi$ is some unknown univariate link function; ${\theta }_{0}\in \mathbb {R}^d$ are coefficients; and $\Delta _0\in \mathbb {R}$ is the potential deviation in baseline risk between the source and target population. We refer to $V = v(X, {\theta }_{0}) = {\theta }_{0}^{T}X$ as *single index*. Note that the intercept for $S = 1$ is absorbed in $\varphi$. For identifiability, let $\Vert {\theta }_{0}\Vert _\infty = 1$ or let its first element be 1.

The invariance in (6) lies in a shared single index and an equal-effect assumption, under which covariates and unmeasured confounding act through the same link function in both populations, with source–target differences represented through a baseline shift. This single index formulation offers a semi-parametric compromise between flexibility and tractability by preserving the low-dimensional summary ${\theta }_{0}^{T}X$ while avoiding a prespecified parametric link. A misspecified parametric link may distort this shared structure, whereas a data-driven link can offer greater robustness. When the link is specified, (6) reduces to the corresponding parametric model, e.g., logistic regression, with common covariate effects across populations but different intercepts. Because the target data are unlabeled, however, the adequacy of (6) cannot be directly assessed from the observed target sample, so its plausibility should be justified primarily by substantive knowledge and scientific considerations.

The single index model (6) provides an additional benefit in dimensionality reduction as the outcome now relies on the covariates only through the univariate single index: $Y \perp \!\!\! \perp X| V, S$. Therefore, it suffices to compute the weights based on *V* since


\begin{eqnarray*}
&&\mathbb {E}\left[w_X\left(X\right) g\left({\theta }_{0}^{T}X\right)| S = 1\right] = \int \frac{f_{X, \mathrm{tg}}(x)}{f_{X, \mathrm{sr}}(x)} g\left({\theta }_{0}^{T}x\right) f_{X, \mathrm{sr}}(x) dx \\&&\quad = \mathbb {E}\left[ g\left({\theta }_{0}^{T}X\right)| S = 0\right] = \mathbb {E}[g(V)| S = 0]\\&&\quad = \mathbb {E}[w_V\left(V\right) g(V)| S = 1] \\&&\quad = \int \frac{f_{V,\mathrm{tg}}(v)}{f_{V,\mathrm{sr}}(v)} g(v) f_{V,\mathrm{sr}}(v) dv
\end{eqnarray*}

for any measurable function *g*. By tracking the data shift through the single index, we avoid difficult high-dimensional non-parametric estimation of model (4). Denote $f_{V,\mathrm{sr}}$ and $f_{V,\mathrm{tg}}$ as densities of the single index, we can replace the previous $w_X$ on *X* by


(7) \begin{eqnarray*}
w_V\left(v\right) &{:=}&\frac{f_{V,\mathrm{tg}}(v)}{f_{V,\mathrm{sr}}(v)} = \frac{(1 - \psi _v\left(v\right)) \mathbb {P}\left(S = 1\right)}{\psi _v\left(v\right) \mathbb {P}\left(S = 0\right)} \text{ for } \psi _v\left(v\right) \\&{:=}&\mathbb {P}\left(S = 1| V = v\right)
\end{eqnarray*}

and $v = v(x, {\theta }_{0}) = {\theta }_{0}^{T}x$. For added flexibility, one can replace the linear single index by an arbitrary pre-specified function (see, e.g., Ichimura, 1993), but this is outside the scope of this paper.

Under model (6), suppose $\mathbb {P}\left(Y = 1| S = 0\right)$ is known; the baseline shift $\Delta _0$ is obtained by solving:


(8) \begin{eqnarray*}
\mathbb {P}\left(Y = 1| S = 0\right) &=& \int _\mathcal {X}\varphi \left(\Delta _0+ {\theta }_{0}^{T}x\right) w_X\left(x\right) f_{X, \mathrm{sr}}(x) dx \\ &=& \int _\mathcal {V}\varphi \left(\Delta _0+ v\right) w_V\left(v\right) f_{V,\mathrm{sr}}(v) dv.
\end{eqnarray*}

For simpler notation, unless otherwise specified, we use $w_{Y}$ (instead of $w_{Y|V}$ as in $w_{Y|X}$) for the $Y|X$-ratio based on *v*, defined by


(9) \begin{eqnarray*}
&& w_{Y}\left(v(x, {\theta }_{0}), y\right) {:=}\frac{\varphi \left(\Delta _0+ v\right)}{\varphi \left(v\right)} \text{ if } y = 1,\\&& \text{ otherwise }\frac{1 - \varphi \left(\Delta _0+ v\right)}{1 - \varphi \left(v\right)}
\end{eqnarray*}

Thus, for any $\left(X, Y\right)$ from source, $w_V\left(v(X, {\theta }_{0})\right) \cdot w_{Y}\left(v(X, {\theta }_{0}), Y\right)$ weights it to be representative of the target population. Hence, for an NP classifier, equation (5) is now written as


(10) \begin{eqnarray*}
\alpha \mathbb {P}\left(Y = 0| S = 0\right) &=& \mathbb {E}[\mathbb {1}_{\left\lbrace \eta (X) \ge c \right\rbrace } (1 - Y) w_V\left(v(X, {\theta }_{0})\right) \cdot w_{Y} \\&&\left(v(X, {\theta }_{0}), Y\right)| S = 1],
\end{eqnarray*}

where now $\eta (x) {:=}\varphi (\Delta _0+ {\theta }_{0}^{T}x)$. We thus obtain the optimal rule $R^{*}(x) = \mathbb {1}_{\left\lbrace \eta (x) \ge c^{*} \right\rbrace }$ for the target population only utilizing the labeled source sample $\left(X, Y\right)| S = 1$ (in (6) and (4)), unlabeled target sample $X| S = 0$ (in (4) or (7)), and the marginal prevalence $\mathbb {P}\left(Y=1| S = 0\right)$ (in (8)), but *without* utilizing joint $(X, Y)$-distribution of the target population directly. Under a correctly specified probability model $\mathbb {P}\left(Y = 1| X, S\right)$, such a shift-adjusted rule will be the most accurate rule for the target population while maintaining control for the desired level of classification error.

### 2.2 Estimation

We now describe the estimation steps of the proposed method. We first estimate quantities in (6) semi-parametrically utilizing the single index assumption. The propensity model (7) that accounts for the covariate shift is estimated by a non-parametric regression of the selection variable *S* on the estimated single index. In the end, plugging in corresponding estimates into (8)–(10) provides an estimated baseline shift and an optimal rule for the target population.

Let $(X_i, Y_i, S_i)$ be i.i.d. copies of $(X, Y, S)$ for $i = 1, \dots , n_{\mathrm{sr}}+ n_{\mathrm{tg}}$. For simplicity, write $(X^\mathrm{sr}_{i}, Y^\mathrm{sr}_{i})$ for $(X_i, Y_i)| S_i = 1$, similarly $(X^\mathrm{tg}_{i}, Y^\mathrm{tg}_{i})$ when $S_i = 0$. Also, the sample could be re-indexed so that $S_i = 1$ for $i = 1, \dots , n_{\mathrm{sr}}$ and $S_i = 0$ for $i = n_{\mathrm{sr}}+ 1, \dots , n_{\mathrm{sr}}+ n_{\mathrm{tg}}$. In practice, only unlabeled observations are available for the target population, so the full data ${\cal D}= {\cal D}_{\mathrm{sr}} \cup {\cal D}_\mathrm{tg}$ consists of the labeled source sample: ${\cal D}_\mathrm{sr}=\left\lbrace \left(X^\mathrm{sr}_{i}, Y^\mathrm{sr}_{i}\right): i = 1, \dots , n_{\mathrm{sr}} \right\rbrace$ and the unlabeled target sample: ${\cal D}_\mathrm{tg}= \lbrace X^\mathrm{tg}_{i} : i = n_{\mathrm{sr}}+ 1, \dots , n_{\mathrm{sr}}+ n_{\mathrm{tg}}\rbrace$, as well as the marginal prevalence $\mathbb {P}\left(Y^\mathrm{tg}_{i} = 1\right)$. The task is to estimate the shift-adjustment weights $w_V$ and $w_{Y}$ by plugging in the estimates of $\varphi , \Delta _0, {\theta }_{0}$, and $\psi _v$.

For simplicity, write $\tau (x) {:=}\varphi ({\theta }_{0}^{T}x)$ in analogy to $\eta$. We first estimate $\varphi$ and ${\theta }_{0}$ semi-parametrically utilizing the source sample $\lbrace (X^\mathrm{sr}_{i}, Y^\mathrm{sr}_{i}): i = 1, \dots , n_{\mathrm{sr}}\rbrace$. Given bandwidth $h_\varphi > 0$, estimate ${\theta }_{0}$ by


(11) \begin{eqnarray*}
\hat{\theta }= \mathrm{arg\, min}_{{\theta }_{0}} \sum _{i=1}^{n_{\mathrm{sr}}} Q\left(Y^\mathrm{sr}_{i}, \hat{\tau }_i({\theta }, h_\varphi )\right)/n_{\mathrm{sr}},
\end{eqnarray*}

where *Q* is some loss function, e.g. squared loss $Q(y, v) = (y - v)^2$ (Ichimura, 1993), cross-entropy loss $Q(y, v) = - y \log v - (1 - y) \log (1 - v)$ (Klein and Spady, 1993). The term $\hat{\tau }_i({\theta }, h_\varphi )$ is a leave-one-out non-parametric estimate for $\mathbb {E}(Y| {\theta }^{T}X^\mathrm{sr})$ with bandwidth $h_\varphi$. For example,


\begin{eqnarray*}
\hat{\tau }_i({\theta }, h_\varphi ) = \frac{ \sum _{j \not= i} Y^\mathrm{sr}_j K\left({\theta }^{T}\left(X^\mathrm{sr}_{i} - X^\mathrm{sr}_j\right)/h_\varphi \right) }{ \sum _{j \not= i} K\left({\theta }^{T}\left(X^\mathrm{sr}_{i} - X^\mathrm{sr}_j\right)/h_\varphi \right) }, i = 1, \dots , n_{\mathrm{sr}},
\end{eqnarray*}

for some kernel function *K*. Further, the conditional risk is obtained by $\hat{\tau }(x) = \hat{\varphi }(\hat{\theta }^{T}x)$ based on the estimated index $\hat{v}_i {:=}\hat{\theta }^{T}X^\mathrm{sr}_{i}$ for $i = 1, \dots , n_{\mathrm{sr}}$ following


(12) \begin{eqnarray*}
&& \hat{\varphi }\left(v\right) = \frac{ \sum _{j=1}^{n_{\mathrm{sr}}} Y^\mathrm{sr}_j K\left(\left(v - \hat{v}_j\right) / h_\varphi \right) }{ \sum _{j=1}^{n_{\mathrm{sr}}} K\left(\left(v - \hat{v}_j\right) /h_\varphi \right) }, \\&&\mathrm{for}\,\, v \,\, \text{in some neighborhood} \,\mathcal {V}\,\mathrm{of} \,\left\lbrace \hat{v}_1, \dots , \hat{v}_{n_{\mathrm{sr}}} \right\rbrace .
\end{eqnarray*}

Next, $w_V$ is estimated by $\hat{w}_V= \left(n_{\mathrm{sr}}\left(1 - \hat{\psi }_v\right)\right) / \left(n_{\mathrm{tg}}\hat{\psi }_v\right)$, where


(13) \begin{eqnarray*}
\hat{\psi }_v\left(v\right) = \frac{ \sum _{j=1}^{n_{\mathrm{sr}}+n_{\mathrm{tg}}} S_j K\left(\left(v - \hat{v}_j\right) / h_\psi \right) }{ \sum _{j=1}^{n_{\mathrm{sr}}+n_{\mathrm{tg}}} K\left(\left(v - \hat{v}_j\right) / h_\psi \right) } \text{ for}\quad v\,\,\, \in \mathcal {V}
\end{eqnarray*}

estimates the propensity $\mathbb {P}\left(S = 1| V = v\right)$ constructed upon pooling $\hat{v}= \hat{\theta }^{T}x$ from both the source and target samples.

Similarly, a plug-in estimator $\hat{w}_{Y}$ estimates $w_{Y}$ by replacing $\varphi , \Delta _0$, and ${\theta }_{0}$ in (9) by corresponding estimations. The estimated baseline shift ${\hat{\Delta }_0}$ is computed via solving for $\Delta$ an empirical version of (8) as


(14) \begin{eqnarray*}
\mathbb {P}\left(Y^\mathrm{tg}= 1\right) = \frac{1}{n_{\mathrm{sr}}}\sum _{i = 1}^{n_{\mathrm{sr}}} \hat{\varphi }\left(\Delta + \hat{\theta }^{T}X^\mathrm{sr}_{i}\right) \hat{w}_V\left(\hat{\theta }^{T}X^\mathrm{sr}_{i}\right).
\end{eqnarray*}

In the end, $\hat{w}_i = \hat{w}_V(\hat{\theta }^{T}X^\mathrm{sr}_{i}) \hat{w}_{Y}(\hat{\theta }^{T}X^\mathrm{sr}_{i}, Y^\mathrm{sr}_{i})$ for $i = 1, \dots , n_{\mathrm{sr}}$ reweight the source sample to be representative of the target population. Subsequently, the NP classifier can be obtained by plugging-in: $\hat{R}(x) = \mathbb {1}_{\left\lbrace \hat{\eta }\left(x\right) \ge \hat{c}_\alpha \right\rbrace }$, where $\hat{\eta }(x) = \hat{\varphi }({\hat{\Delta }_0}+ \hat{\theta }^{T}x)$, while $\hat{c}_\alpha$ solves the empirical version of (10) as


(15) \begin{eqnarray*}
\hat{c}_\alpha = \inf \left\lbrace c: \frac{ \sum _{i=1}^{n_{\mathrm{sr}}} \mathbb {1}_{\left\lbrace \hat{\eta }\left(X^\mathrm{sr}_{i}\right) \ge c \right\rbrace } \hat{w}_i \left(1 - Y^\mathrm{sr}_{i}\right) }{ \sum _{i = 1}^{n_{\mathrm{sr}}} \hat{w}_i \left(1 - Y^\mathrm{sr}_{i}\right) } \le \alpha \right\rbrace .
\end{eqnarray*}

For a simpler theoretical treatment, data are usually split so that observations used for (15) are independent of those used to estimate other quantities. We also dedicate a part of the data for estimating $\hat{\theta }$ to tackle bias due to the estimated single index. We detail such a data split procedure in Subsection S4.1 of the Supplement. However, it appears unnecessary and potentially wasteful, especially given the smaller sample. Thus, our implementation uses all observations.

## 3 Consistency and rate of convergence

We show that the proposed SACSIM weighting consistently estimates the data shift and that the plug-in NP classifier (15) asymptotically achieves the desired FPR control while attaining the optimal TPR for the target population. The main results are summarized here, with assumptions and proofs provided in Sections S4–S7 of the Supplement.

As detailed in Section S4, conditions [D1]–[D3], (A1)–(A5), (P1)–(P3), and (K1) impose standard regularity, overlap, margin, sample-size, and kernel-smoothing requirements. In particular, they require the relevant density functions and $\mathbb {P}\left(Y=1\mid X\right)$ to be smooth and well-behaved, the source and target covariate distributions to have sufficient overlap so that the density ratios are well defined, and the parameters ${\theta }_{0}$ and $\Delta _0$ to lie in neighborhoods where consistent estimation is possible. They also ensure that the optimal cutoff $c_\alpha ^{*}$ exists and is estimable under standard margin and detection conditions for plug-in NP classifiers (see, e.g., Tong, 2013). The sample-size condition requires both components, the source data and the unlabeled target data, to grow with the total sample size, so that the rates can be stated compactly in terms of *n*. Under these conditions, the kernel-smoothed components are uniformly consistent, which yields the following rate for the estimated baseline shift.

Proposition 3.1: Under conditions (A1)–(A5), [D1]–[D3], and (K1), suppose the bandwidths $h_\varphi$ and $h_\psi$ in (12) and (13) shrink to zero as $n\rightarrow \infty$ such that

\begin{eqnarray*}
&& \max \left(h_\varphi , h_\psi \right) \rightarrow 0, n h_\psi ^2 \rightarrow \infty , \log n / (n h_\psi ) \rightarrow 0,\\&& \left|\log h_\varphi \right| / \left({n}h_\varphi ^4\right) \rightarrow 0,\quad \text{and } {n}h_\varphi ^8 \rightarrow 0,
\end{eqnarray*}

then $\left|{\hat{\Delta }_0}- \Delta _0\right| = O_p ( b_{n})$, where $b_{n}= h_\psi ^2 + 1/\left(\sqrt{n}h_\psi \right) + \sqrt{\log {n}/ ({n}h_\psi )} + h_\varphi ^2 + 1/\left(\sqrt{n}h_\varphi \right)$.

The rate in Proposition 3.1 combines the Z-estimation error for $\Delta _0$ with the uniform rates of the plug-in estimators of $\varphi$, ${\theta }_{0}$, and $w_V$ in (14). Because the single index ${\theta }_{0}^{T}X$ is unknown and replaced by $\hat{\theta }^{T}X$, the proof also accounts for this error-in-variable effect, following arguments similar to Chiou and Müller (1998) under $\left\Vert \hat{\theta }-{\theta }_{0}\right\Vert =O_p(n^{-1/2})$ (e.g., Ichimura, 1993). Consequently, the rate is slower than the usual uniform kernel-smoothing rate $h^2+\sqrt{\log n/(nh)}$ (see, e.g., Hansen, 2008), and the propensity-estimation terms in $b_{n}$ reflect the size of the unlabeled target sample.

We now quantify the excess risk of the plug-in shift-adjusted classifier. For any rule *R*, let $\mathrm{TPR}(R)$ and $\mathrm{FPR}(R)$ denote its TPR and FPR on the target population, and define $\mathrm{TPR}^{*} {:=}\max _{R} \mathrm{TPR}(R)$ subject to $\mathrm{FPR}(R)\le \alpha$. Thus, $\mathrm{TPR}^{*}$ is the optimal target-population TPR achievable under the FPR constraint.

Theorem 3.1: Under conditions (P1)–(P3) and those of Proposition 3.1, we have

\begin{eqnarray*}
\left|\mathrm{FPR}(\hat{R}) - \alpha \right| = O_p\left( b_{n}\right) , \quad \left|\mathrm{TPR}(\hat{R}) - \mathrm{TPR}^{*}\right| = O_p\left( b_{n}^ {\min \left\lbrace 1, \frac{1 + \bar{\gamma }}{\underline{\gamma }} \right\rbrace } \right),
\end{eqnarray*}

where $\hat{R}(x) = \mathbb {1}_{\left\lbrace \hat{\eta }(x) > \hat{c}_\alpha \right\rbrace }$ is the estimated classifier with plugged-in conditional risk $\hat{\eta }$ and cutoff $\hat{c}_\alpha$ as in (15); $b_{n}$ is defined in Proposition 3.1; $\bar{\gamma }$ and $\underline{\gamma }$ are from (P2) and (P3); and the $O_p$ notation is with respect to the data sampling distribution.

Theorem 3.1 shows that the achieved FPR of the proposed classifier approaches the target level $\alpha$, while its TPR approaches the optimal constrained value $\mathrm{TPR}^{*}$. The asymptotically optimal bandwidths are $h_\varphi ^{*} \asymp h_\psi ^{*} \asymp n^{-1/6}$, giving $b_{n}^{*} \asymp n^{-1/3}$. This corresponds to oversmoothing relative to the conventional bandwidth $n^{-1/5}$, due to the error-in-variable effect from estimating the single index.

The theoretical derivation uses a three-way data split to compute $\hat{\theta }$, $\hat{c}_\alpha$, and the remaining quantities on separate samples. This differs slightly from the estimation procedure in Subsection 2.2, but is used only to simplify the proof by removing dependence among estimated components in (15). Conditional on the relevant training portions, the empirical FPR can be controlled using VC-type arguments (e.g., Devroye et al., 1996; Tong, 2013), while uniform consistency of kernel-smoothed quantities follows from Talagrand-type inequalities (Giné and Guillou, 2002). Finally, the theory only requires consistent estimation of the risk and propensity components in (6), (4), and (7); it is not tied to the specific local-smoothing estimators used in Subsection 2.2. More general plug-in versions are discussed in Sections S5 and S7.

## 4 Simulation

We now illustrate by simulation the flexibility of the proposed adjustment method. Throughout the experiment, we set ${\theta }_{0}= (1, 1)^{T}$ and $\Delta _0= -0.75$ in (6) so that $\mathbb {P}\left(Y = 1\right)$ was between 0.35 and 0.5. Combining different *X*-shifts with varying link functions in the response model (6) constitutes nine simulation setups. The source $X^\mathrm{sr}$ was drawn from a bivariate normal distribution with $n_{\mathrm{sr}}= 750$, and $Y^\mathrm{sr}$ was drawn under model (6). These data were used to estimate the unadjusted NP classifiers over different levels of control over TPRs or FPRs ($1 - \beta$ and $\alpha$ in problems (2) and (1)) along the grid of $0.05, 0.1, \dots , 0.9$, and 0.95. Those NP classifiers were then adjusted following the proposed SACSIM approach utilizing an unlabeled sample of size $n_{\mathrm{tg}}= 100$ drawn from a different target population. The performance of shift adjustment was evaluated on another independent target sample with a size of 10,000. For simplicity, in the main text we report the mean absolute error (MAE): the absolute discrepancy between the achieved and desired TPR within the target population, averaged over the grid of desired control levels $0.05, \dots , 0.95$. Similarly, we report the gap to the optimal FPR, defined as the MAE between the achieved FPR and the optimal FPR at the corresponding retained TPR level, again averaged over the same grid.

**Figure 1:**
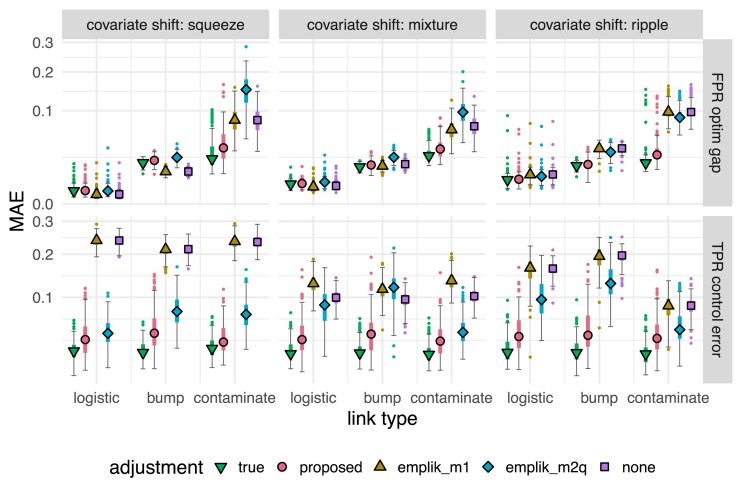
Shift-adjustment performance in terms of controlling the TPR while minimizing the FPR, under weighting by the true ratio, the proposed SACSIM, empirical likelihood (emplik_m1 and emplik_m2q), and no adjustment (none). Large symbols denote medians across 200 repeated experiments; vertical intervals show interquartile ranges and Tukey whiskers. Small points denote outliers beyond the whiskers. Abbreviations: FPR, false positive rate; SACSIM, shift-adjusted Neyman–Pearson classifier via single index modeling; TPR, true positive rate.

For comparison, we consider several approaches for computing the weights in (15): (i) empirical likelihood that matches the sample statistics (detailed in Section S2 of the Supplement), (ii) using the true $w_Xw_{Y|X}$, and (iii) unadjusted setting all weights to 1. We repeated each simulation setup 200 times and summarized the results in Figure 1.

Figure 1 shows that naively borrowing an unadjusted rule across populations can severely degrade performance, where the average error in control could be as high as 25%. In contrast, SACSIM delivers the smallest control error among practical methods, with performance closest to the oracle adjustment based on the true weights. This indicates that the proposed weighting scheme effectively mitigates the impact of data shift and prevents excessive violations of the desired error-control target. Empirical likelihood reweighting, however, does not reliably preserve control. Matching only sample means (emplik_m1) is often insufficient, and while incorporating additional constraints, such as second moments and quantiles (emplik_m2q), can help in some settings, improvements are not uniform. For example, under the bump-type $\varphi$ with mixture *X*-shift, even the richer set of constraints can fail to achieve stable control. As discussed in Section S2 of the Supplement, the empirical likelihood-based adjustment relies on correctly specifying structural constraints to relate the source and target populations. In practice, these constraints are hard to justify without substantial prior knowledge of the target distribution. This sensitivity limits its utility for adjusting NP classifiers under data shift.

Finally, SACSIM is closest to the truth not only in enforcing the desired TPR control, but also in achieving near-optimal FPR at the retained TPR level. In contrast, some alternatives can appear to have a smaller MAE in the FPR optimal gap, but this comparison can be misleading: their smaller FPR gap is often driven by failing to retain the intended TPR level (i.e., a larger MAE in TPR control), so the FPR is being evaluated at an effectively different, and typically easier, operating point. When the desired TPR is actually maintained, SACSIM yields FPR performance that is consistently closest to the truth across the grid of control levels.

Additional simulation details, together with robustness studies under mild violations of the equal-effect assumption in (6), are provided in Section S9 of the Supplement.

## 5 Example

In prostate cancer, while the early detection and treatment of aggressive diseases offer survival benefits, broad screening initiatives often result in the overdiagnosis of low-risk individuals who may not require an invasive diagnostic biopsy or surgical procedure. In the PCA3 Evaluation Trial (PCA3 trial) from the National Cancer Institute Early Detection Research Network (e.g., Tosoian et al., 2024; Wei et al., 2014), 743 participants entered the study with signs of an elevated risk of prostate cancer. All participants subsequently received a diagnostic biopsy, 151 of whom had an adverse outcome of high-grade cancer (Gleason score $\ge$ 7). In this setting, due to the invasive nature of the prostate cancer diagnostic biopsy, personalized decision rules based on noninvasive clinical and biomarker information are needed to rule out low-risk patients for a diagnostic biopsy or rule in high-risk patients for more aggressive procedures, balancing risk and benefit in each scenario. For illustration, we consider building NP rules using four covariates: age, serum PSA, a urine RNA marker score, and an indicator of prior negative biopsy.

The PCA3 trial predominantly included White patients, comprising 596 participants (80%), while Black patients represented a smaller group with 94 participants (13%) and 53 patients from other races (7%). Given the known racial disparity in prostate cancer characteristics and outcomes (Hinata and Fujisawa, 2022), one crucial question is whether the rules derived in this cohort attain equal risk across diverse populations.

**Figure 2:**
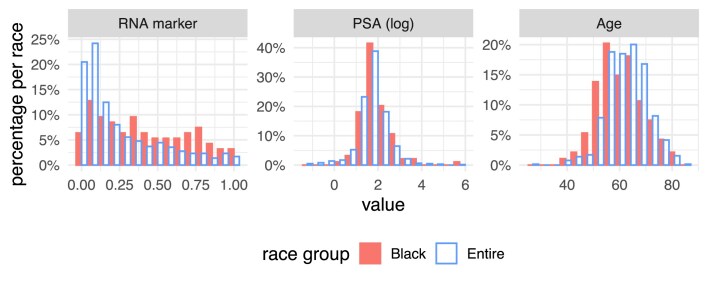
Histograms of the RNA marker, PSA (log scale), and age of the Black population vs. the entire population.

Table 1 and Figure 2 summarize data distributions by different racial groups, which suggest, in general, Black patients are younger yet have higher biomarker values and more unfavorable outcomes. Such disparity may cause a considerable error when a rule learned from the entire population is applied unadjusted to the less representative population, as shown in Table 2. For example, when an NP classifier requiring an FPR of no more than 0.3 was initially derived using data from the entire population and applied to the Black population, the FPR exceeded 0.53, potentially leading to substantially more unnecessary invasive procedures.

**Table 1:** Summary statistics (Sample mean, standard deviation (SD), minimum (Min), first quartile (Q1), median, third quartile (Q3), and maximum (Max)) for the outcome and covariates of the PCA3 data.

Population	*P*-value	Mean	SD	Min	Q1	Median	Q3	Max
**Outcome: Gleason $\ge$ 7**								
Black	0.30	0.26	–	–	–	–	–	–
White	0.58	0.19	–	–	–	–	–	–
Other	0.38	0.26	–	–	–	–	–	–
Entire	–	0.20	–	–	–	–	–	–
**RNA marker**								
Black	4e-07	0.42	0.29	0.01	0.16	0.37	0.64	1.00
White	0.14	0.25	0.27	0.00	0.05	0.13	0.39	1.00
Other	0.63	0.25	0.27	0.00	0.06	0.14	0.34	0.99
Entire	–	0.27	0.28	0.00	0.05	0.15	0.43	1.00
**PSA (log scale)**								
Black	0.03	1.94	0.70	0.41	1.53	1.84	2.26	5.71
White	0.48	1.72	0.73	−1.24	1.41	1.70	2.05	5.73
Other	0.95	1.77	0.70	−0.92	1.44	1.70	1.99	3.46
Entire	–	1.75	0.73	−1.24	1.41	1.72	2.08	5.73
**Age**								
Black	0.09	60.93	8.39	41.00	55.00	60.50	66.00	82.00
White	0.58	62.44	7.88	27.00	57.00	63.00	68.00	85.00
Other	0.91	62.34	5.81	52.00	58.00	62.00	67.00	75.00
Entire	–	62.24	7.83	27.00	57.00	62.00	68.00	85.00
**Has prior negative biopsies**								
Black	0.24	0.27	–	–	–	–	–	–
White	0.75	0.34	–	–	–	–	–	–
Other	1.00	0.34	–	–	–	–	–	–
Entire	–	0.33	–	–	–	–	–	–

*P*-values are for comparing each subpopulation distribution to that of the entire population by the Wilcoxon rank-sum test for continuous variables and $\chi ^2$ test for binary variables. Abbreviation: SD, standard deviation.

**Table 2:** Performance of rules learned from the entire population applied to the Black population, SACSIM-adjusted or unadjusted.

Desired		Absolute	Achieved	
TPR	FPR	Error	TPR (95% CI)	FPR (95% CI)
**SACSIM**				
–	0.1	0.016	0.51 (0.37, 0.64)	0.10 (0.07, 0.14)
–	0.2	0.017	0.68 (0.54, 0.80)	0.19 (0.16, 0.23)
–	0.3	0.027	0.76 (0.66, 0.84)	0.32 (0.27, 0.37)
0.7	–	0.049	0.72 (0.59, 0.82)	0.23 (0.18, 0.28)
0.8	–	0.038	0.79 (0.69, 0.88)	0.36 (0.30, 0.42)
0.9	–	0.040	0.88 (0.78, 0.97)	0.48 (0.43, 0.54)
**none**				
–	0.1	0.097	0.69 (0.57, 0.80)	0.20 (0.17, 0.23)
–	0.2	0.207	0.83 (0.73, 0.91)	0.41 (0.36, 0.46)
–	0.3	0.235	0.92 (0.83, 1.00)	0.53 (0.49, 0.58)
0.7	–	0.131	0.83 (0.73, 0.92)	0.41 (0.36, 0.47)
0.8	–	0.094	0.89 (0.81, 0.97)	0.50 (0.44, 0.55)
0.9	–	0.077	0.97 (0.88, 1.00)	0.69 (0.63, 0.75)

Averages of achieved TPR and FPR and 95% confidence intervals (95% CI) at TPR or FPR controlled at 0.1, 0.2, 0.3, or 0.7, 0.8, and 0.9, respectively, are listed, based on 50 repeated 5-fold cross-validations. Abbreviations: FPR, false positive rate; TPR, true positive rate.

We adjusted the decision rules following the proposed SACSIM method to ensure they provide similar error controls for the Black group. We conducted 200 repeated experiments of 5-fold cross-validation. In each repeated experiment, the data were randomly partitioned into 5-folds, 4 of which were used to derive the NP classifiers. They were then adjusted for data shift utilizing the disease prevalence and the covariate information (the four covariates listed in Table 1) of the corresponding racial groups (White, Black, or entire), with performance assessed on the held-out fold. See Section S10 in the Supplement.

**Figure 3:**
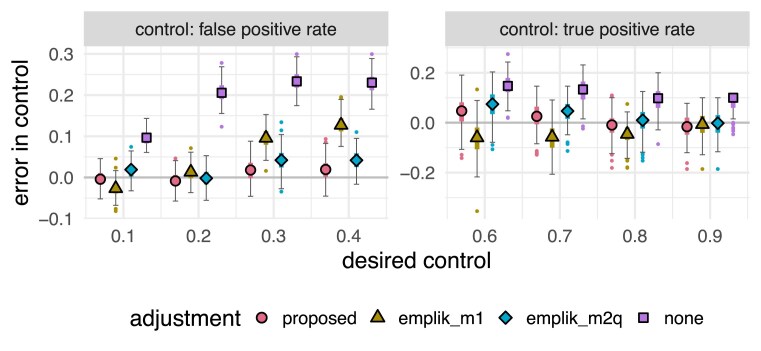
The errors (achieved minus expected) in control over different levels of control (*x*-axis) over TPR (right panel) or FPR (left panel) when applying rules learned from the entire population to the Black population, subject to different adjustments. Abbreviations: FPR, false positive rate; TPR, true positive rate.

**Figure 4:**
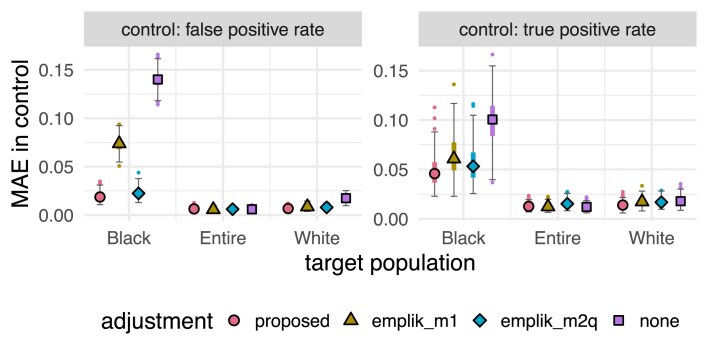
The MAE (averaging all absolute errors over a grid of control levels of 0.01, 0.05, 0.1, 0.15,..., 0.9, 0.95, 0.99) in control over TPR (right panel) or FPR (left panel), when applying rules learned from the entire population to the entire, Black, or the White population, subject to different adjustments. Abbreviations: FPR, false positive rate; MAE, mean absolute error; TPR, true positive rate.

Compared to estimates without any adjustment, the proposed adjustment method maintained the desired TPR or FPR control in the Black population, as shown in Table 2. To further investigate the reduction in error, we compared the SACSIM method to the empirical likelihood approach, which adjusts based on moments and quantiles (for details, see Section S2 of the Supplement). As shown in Figure 3, emplik_m1 yielded only limited improvement and at times failed to maintain the desired control. The richer emplik_m2q specification performed competitively and was often close to SACSIM in this example, likely because the imposed summaries, including first two moments and multiple quantiles, were already highly informative about the target covariate distribution. This illustrates that empirical likelihood adjustment can work when sufficiently rich target summaries are available. However, even in this favorable setting, SACSIM still tended to yield slightly smaller errors overall, without requiring the analyst to prespecify and acquire a large set of summary statistics. This same pattern appears in Figure 4: relative to the unadjusted approach and emplik_m1, both SACSIM and emplik_m2q markedly reduced MAE for the Black population, with SACSIM achieving the smallest overall MAE.

## 6 Summary

The NP classifier is widely applicable in decision-making contexts where benefits must be balanced against potential harms. However, its effectiveness can be limited when applied to a new population that is underrepresented in the original training cohort due to its sensitivity to data shifts between the target and source populations. This mismatch can lead to unintended consequences and reduced performance. To address this challenge, enhancing the classifier’s ability to generalize across diverse groups is crucial, minimizing reliance on specific statistical assumptions and ensuring consistent outcomes. To this end, we propose an adjustment methodology to improve the transportability of NP classifiers across heterogeneous populations. We focus on achieving equal risk control of the NP classifier between the source and target populations by controlling a common level of acceptable harm while maximizing the benefits at the acceptable level of risk for each population.

We adopt a single-index differential risk model for $\mathbb {P}\left(Y|X,S\right)$, which not only accounts for $Y|X$-shift but also addresses *X*-shift by reducing dimensionality through the single-index component. This approach mitigates the need for strong modeling assumptions, offering greater flexibility while maintaining the interpretability of predictions. The model’s flexibility enhances the robustness of the density ratio estimates, ensuring the NP-classifier’s consistency for the target population. For estimation, direct observations of the joint distribution of $(X, Y)$ in both populations are typically needed. While such information is often available in the study cohort from the source population, it is usually not accessible in specific subpopulations. In many practical settings, such as in prostate cancer diagnosis and surveillance, the ascertainment of a definite outcome *Y* at the individual level often requires more effort and may not be readily available, including long-term follow-up and expensive diagnostic procedures, and is prone to misclassification errors or verification bias. Our proposed approach accommodates the scenario in which the target population provides only limited data: a small sample of covariates along with information about the marginal disease prevalence $\mathbb {P}\left(Y=1\right)$. When the unlabeled samples cannot be directly shared due to privacy or policy constraints, the requirement could be relaxed if the relevant covariate distributions were available through privacy-preserving representations, such as synthetic data or distributional summaries. Finally, our method for estimating the joint selection probability can be used as a general framework to adjust for data shift for other consistent estimating approaches, ensuring the generalizability, applicability, and trustworthiness of clinical rules applied to diverse populations.

We assume identical covariate effects between the source cohort and the target population. While this assumption is generally considered acceptable in practice, one must carefully weigh this assumption based on domain knowledge and existing epidemiological evidence regarding the stability of covariate effects across related populations. When individual-level linked data from the target population are available, formal tests comparing regression coefficients can be performed. However, such data are typically unavailable in recalibration settings, as their availability would obviate the need for recalibration.

Several alternative frameworks have been proposed in the literature. For instance, Tan et al. (2022) and Sahoo et al. (2023) suggest learning a single decision rule for the entire population, minimizing the worst-case risk through distributionally robust optimization to protect vulnerable groups. Each approach has its advantages. Our approach, which customizes the rule for each group, ensures optimal performance tailored to the specific needs of each population. In contrast, the alternative approach offers the simplicity and practical appeal of using a single decision rule for all groups, making it easier to implement. In clinical settings, practitioners can choose between these two methods depending on their priorities: optimal performance for each population or ease of implementation across the board.

## Supplementary Material

ujag138_Supplemental_Files Web Appendices including additional simulation studies and proofs of the theoretical results, referenced in Sections 2–5, and the code are available with this paper at the Biometrics website on Oxford Academic.

## Data Availability

The PCA3 data used in this article are not publicly available due to data use agreements. The R code implementing the proposed method are available in the Supplementary Materials.
