# Supplementary material for: Shift-adjusted Neyman–Pearson classifiers via single index modeling (SACSIM)
Source: Biometrics. 2026 Aug 10;82(3):ujag138. doi: 10.1093/biomtc/ujag138 (PMC13455594; doi:10.1093/biomtc/ujag138)
Supplement: Supplementary file 1 [file ujag138_supplemental_files.zip › shiftAdjCode_public/shiftAdjCode/simuCode/fig/simu/simu_link.pdf]

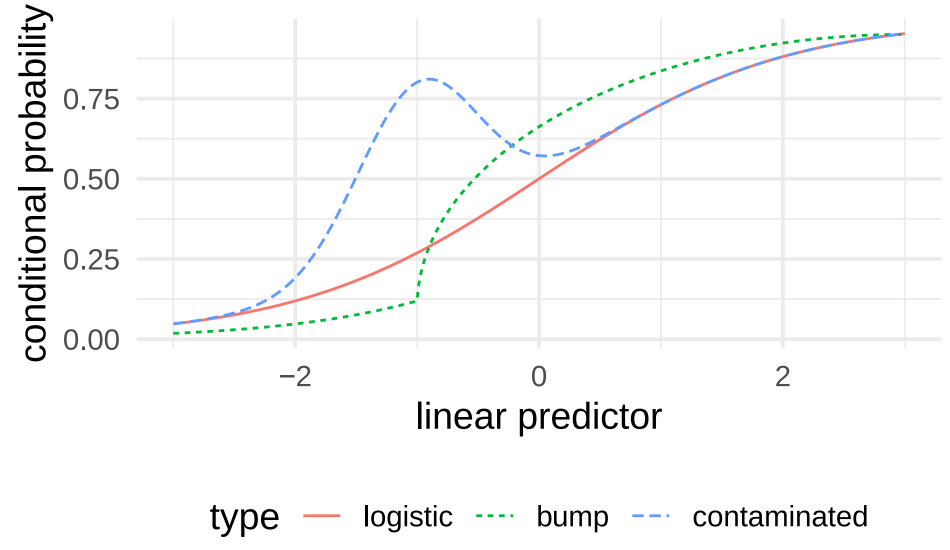
conditional probability
0.00
0.25
0.50
0.75
−2
0
2
linear predictor
type
logistic
bump
contaminated
