# Supplementary material for: Shift-adjusted Neyman–Pearson classifiers via single index modeling (SACSIM)
Source: Biometrics. 2026 Aug 10;82(3):ujag138. doi: 10.1093/biomtc/ujag138 (PMC13455594; doi:10.1093/biomtc/ujag138)
Supplement: Supplementary file 1 [file ujag138_supplemental_files.zip › shiftAdjCode_public/shiftAdjCode/simuCode/fig/simu/simu_tfp_capped_tukey_outliers_tp_1000.pdf]

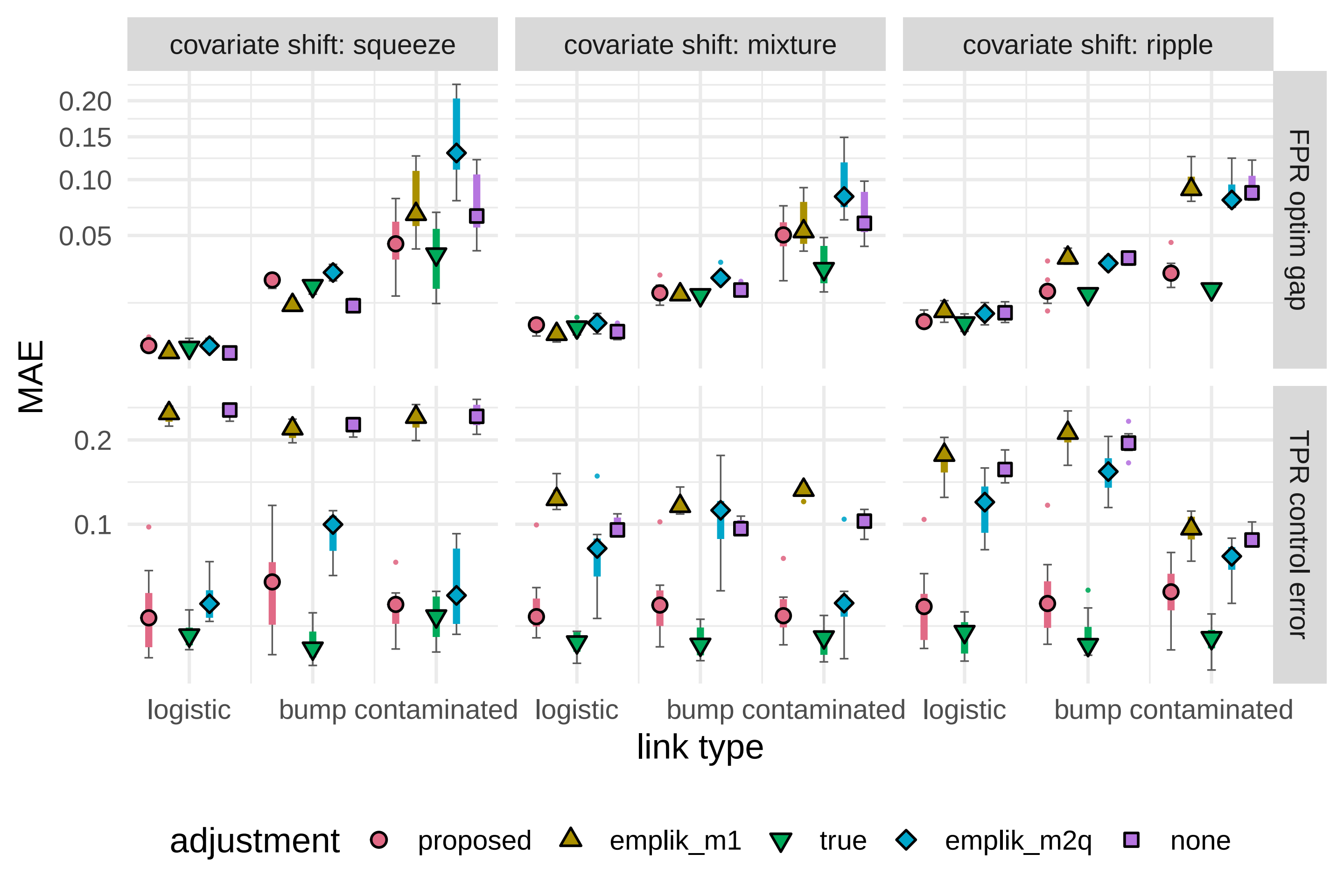

covariate shift: squeeze
covariate shift: mixture
covariate shift: ripple
FPR optim gap
TPR control error
MAE
0.20
0.15
0.10
0.05
0.2
0.1
logistic
bump contaminated
link type
adjustment
proposed
emplik_m1
true
emplik_m2q
none
