# Supplementary material for: Shift-adjusted Neyman–Pearson classifiers via single index modeling (SACSIM)
Source: Biometrics. 2026 Aug 10;82(3):ujag138. doi: 10.1093/biomtc/ujag138 (PMC13455594; doi:10.1093/biomtc/ujag138)
Supplement: Supplementary file 1 [file ujag138_supplemental_files.zip › surveSupp.pdf]

Supplementary Materials for "Shift-Adjusted Neyman–Pearson Classifiers via Single Index Modeling (SACSIM)"

Jiaming Qiu[1], Ying-Qi Zhao[1], and Yingye Zheng[1]

[1] *Public Health Division, Fred Hutchinson Cancer Center*

Contents

S1 Data-shift Affecting Classification **2**

S2 Empirical Likelihood **2**

S3 Preliminaries for Theoretical Developments **4**
- S3.1 Optimality of Likelihood Rules . . . 4
- S3.2 Vapnik–Chervonenkis Inequality . . . 5
- S3.3 Justifying Weights on the Single Index . . . 7

S4 List of Assumptions **9**
- S4.1 Data Split and Growth in Sizes . . . 10

S5 Consistency of True/False Positive Rate **11**

S6 Uniform Consistency of Nadaraya–Watson Estimator **18**

S7 Analysis of the Plug-in Estimators **20**
- S7.1 Single Index Model . . . 20
- S7.2 Estimated Baseline Shift . . . 21
- S7.3 Proof of Theorem 3.1 . . . 23

S8 Implementation Notes **24**

S9 Simulation Details **24**
- S9.1 Logistic regression vs single index model . . . 26
- S9.2 Computation time . . . 26
- S9.3 Impact of Assumption Violation . . . 30

S10 PCA3 Data **33**

S1 Data-shift Affecting Classification

Example S1.1. Consider a univariate logistic regression example with three populations: source, target populations with either $Y|X$-shift or both shifts. The covariate for the former two follows $N(0, 2)$, i.e., normal distribution with mean zero and variance 2, while for the both-shift population, the covariate follows $N(0, 1)$, the standard normal distribution. The risk follows $\mathbb{P}(Y = 1|X) = \text{logistic}(X)$ for the source population, while for the latter two populations $\mathbb{P}(Y = 1|X) = \text{logistic}(1 + X)$. As the risk functions are monotone increasing functions of X, the NP classifiers take the form of $\mathbb{1}_{\{X \geq c\}}$ for some threshold c. Benchmarking NP classifiers on these populations illustrates the effects of $Y|X$-shift (source vs. $Y|X$-shift), X-shift ($Y|X$-shift vs. both-shift), and combined (source vs. both-shift), as pictured in Figure S1.1.

The $Y|X$-shift shares the same receiver operating characteristic (ROC) curve as the source since the ranking of the subjects is invariant; yet the same cutoff can correspond to different points on the curve, therefore still damaging if neglected. For example, the TPR of the classifier $\mathbb{1}_{\{x \geq -1\}}$ drops to 0.89 from 0.93 under $Y|X$-shift. Consequently, a classifier could fail to reach the desired TPR should such a $Y|X$-shift exist between the source and target population.

We now further compare the two populations of "$Y|X$-shift" (triangles) and "both-shift" (squares), where only the covariate is distributed differently (i.e., X-shift). Observe that such an X-shift deforms the ROC curve. Suppose $\mathbb{1}_{\{x \geq -1\}}$ was chosen based on the "$Y|X$-shift" population with the expectation of barring FPR below 0.5, the actual FPR could be 0.67 in the "both-shift" population as a consequence of the X-shift.

S2 Empirical Likelihood

Owen (1990) proposed the empirical likelihood framework. Given sample $X_1, \ldots, X_n$, it seeks weights (i.e., *empirical likelihood* of each observation) $w_1^*, \ldots, w_n^*$ that maximizes $\sum_i \log w_i$ subject to

$$\sum_i w_i g(X_i) = 0, \sum_i w_i = 1, \text{ and } w_i > 0. \tag{S2.1}$$

for some structural constraint function $g : \mathcal{X} \to \mathbb{R}^p$. It has been adopted to improve the efficiency of parameter estimates under covariate shift (Han and Lawless, 2019; Zheng et al., 2021), where the model likelihood is maximized subject to structural constraints obtained from auxiliary samples to borrow information.

Constructed by constrained optimization, the empirical likelihood can be intuitively understood as sample weights that "align" the statistics across the source and target population. For example, weights generated on $X_1^{\text{sr}}, \ldots, X_{n_{\text{sr}}}^{\text{sr}}$ by setting $g(x, \theta) = x - \mathbb{E}(X^{\text{tg}})$ from (S2.1) forces the weighted average of source sample to be the expectation of the target population: $\sum_i w_i^* X_i^{\text{sr}} = \mathbb{E}[X^{\text{tg}}]$. After reweighting, the alignment of sample statistics makes the source sample appear more representative of the target population. Thus, it could be tempting to adopt the empirical likelihood directly as a sample weight and subsequently draw inferences based on the reweighted source sample, as explored by Zheng et al. (2021); Chu et al. (2022).

However, empirical likelihood is not a consistent estimate for the true sample weight $\mathbb{P}(X, Y|S = 0) / \mathbb{P}(X, Y|S = 1)$ except under special situations where the structure constraints coincide with the covariate distribution (e.g., Chu et al., 2022, section 3.2), as it is not difficult to see that $w_i^* = \left(1 - \lambda^T g(X_i)\right)^{-1}$ for some λ. While it is shown that some specific structural constraints help to reduce the Kullback–Leibler (KL) divergence to the target distribution after empirical likelihood reweighting (Zheng et al., 2021), the lack of consistency and strong dependency on the choice of structural constraints makes it unsuitable for adjusting NP classifiers, which is also evident from our simulation and real data examples.

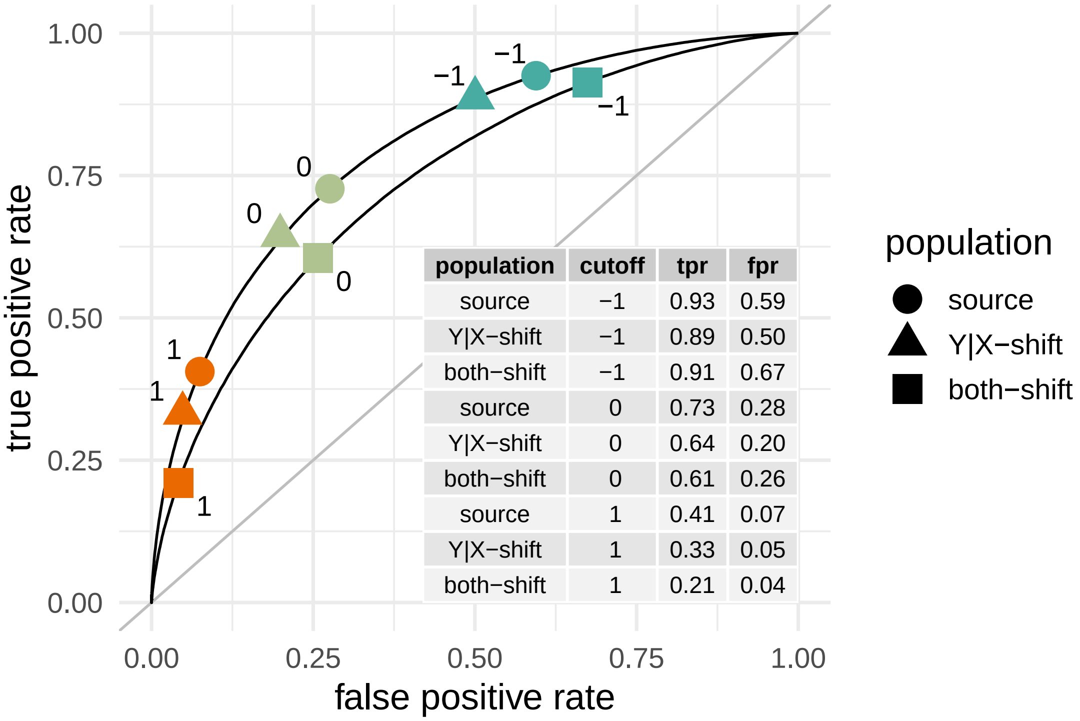

population	cutoff	tpr	fpr
source	−1	0.93	0.59
Y|X−shift	−1	0.89	0.50
both−shift	−1	0.91	0.67
source	0	0.73	0.28
Y|X−shift	0	0.64	0.20
both−shift	0	0.61	0.26
source	1	0.41	0.07
Y|X−shift	1	0.33	0.05
both−shift	1	0.21	0.04

Figure S1.1: Effect of data shift on ROC curves of NP classifiers under no data shift (source), only $Y|X$-shift, or both $Y|X$ and X-shift. The embedded table shows NP classifier $\mathbb{1}_{\{x \geq c\}}$ performance corresponding to the annotated dots on the curves, where c is listed as the cutoff.

In our examples we considered two different structural constraints. One matches the sample averages (emplik_m1) by setting $g(x) = x - \mathbb{E}(X^{\mathrm{tg}})$. The other (emplik_m2q) matches the first two moments and the quantiles by setting $g(x) = (g_1(x), \ldots, g_7(x))^T$, with $g_6 = x - \mathbb{E}[X^{\mathrm{tg}}]$ and $g_7 = x - \mathbb{E}[(X^{\mathrm{tg}})^2]$ matching the first two moments, and $g_j = \mathbb{1}_{\{x \le \theta_j\}} - q_j$ with θ_j being the $(j-1)/4$-quantile of X^{tg} for $j = 1, \ldots, 5$. Structural constraints are applied to every covariate in the case of multiple covariates. The resulting empirical likelihood $w_1^*, \ldots, w_{n_{\mathrm{sr}}}^*$ are used as sample weights in, e.g., (15) of the main text.

S3 Preliminaries for Theoretical Developments

S3.1 Optimality of Likelihood Rules

Here we provide further detail to clarify that it suffices to consider likelihood ratio when tackling the optimization problem (1). The following proposition borrows the Neyman–Pearson lemma (see e.g., Neyman et al., 1933; Casella and Berger, 2002).

Proposition S3.1. *Given that* $0 < \mathbb{P}(Y = 1|X) < 1$*, a rule* R^* *is optimal for* (1) *if and only if it satisfies*

$$R^*(X) = \begin{cases} 1, & \text{if } \mathbb{P}(Y = 1|X) > c; \\ 0, & \text{if } \mathbb{P}(Y = 1|X) < c; \end{cases}$$

and $\mathbb{E}(R^*(X)|Y = 0) = \alpha$.

Proof. Note that $\mathbb{P}(Y = 1|X) / \mathbb{P}(Y = 0|X)$ is an increasing function of $\mathbb{P}(Y = 1|X)$. By Bayes rule,

$$\frac{\mathbb{P}(Y = 1|X)}{\mathbb{P}(Y = 0|X)} \propto \frac{\mathbb{P}(X|Y = 1)}{\mathbb{P}(X|Y = 0)}.$$

Use the same argument for the Neyman–Pearson lemma w.r.t. the conditional distribution $X|Y = 0$ and $X|Y = 1$ (treating as null and alternative hypothesis, respectively). □

We can also derive the same result from an optimization perspective utilizing Lagrangian multiplier. The Lagrangian function (see e.g. Boyd and Vandenberghe, 2004, Chapter 5) of the optimization problem (1) is

$$L(R, \lambda) = -\mathbb{E}\left(\mathbb{1}_{\{R(X)=1\}} (\rho_1(X) - \lambda\rho_0(X))\right) - \lambda\alpha,$$

where $\rho_y(X) = \mathbb{P}(Y = y|X) / \mathbb{P}(Y = y)$ for $y = 0, 1$. Its dual function is $g(\lambda) = \inf_R L(R, \lambda)$, where the infimum is obtained at

$$R_\lambda(X) = \mathbb{1}_{\{\rho_1(X) - \lambda\rho_0(X) > 0\}}$$

for $\lambda \ge 0$. Let $\lambda^* > 0$ be the solution to

$$\mathrm{TPR}(R_\lambda) = \mathbb{E}\left(\mathbb{1}_{\{\rho_1(X) - \lambda\rho_0(X) > 0\}} \rho_0(X)\right) = \alpha \tag{S3.1}$$

for $\lambda > 0$ and $0 < \alpha < 1$. Noting that $\mathrm{TPR}(R_{\lambda^*}) = -g(\lambda^*)$ with no duality gap (i.e., strong duality holds), so λ^* is the dual optimal, while R_{λ^*} is the optimal rule. We argue $\lambda^* \ne 0$ since $\mathrm{FPR}(R_\lambda|_{\lambda=0}) = 1 \ne \alpha$. Therefore, the primal problem is equivalently finding λ^* by solving (S3.1). The rule R_{λ^*} is equivalently

$$R_{\lambda^*}(X) = \mathbb{1}_{\{\rho_1(X)/\rho_0(X) > \lambda^*\}},$$

which is a likelihood ratio rule.

S3.2 Vapnik–Chervonenkis Inequality

We now revisit the Vapnik–Chervonenkis (VC) inequality (e.g., Devroye et al., 1996). Given covariate space $\mathcal{X}$ and response $Y \in \mathcal{Y} = \{0, 1\}$, let $\mathcal{F} = \{f : \mathcal{X} \to \mathcal{Y}\}$ be a class of classifiers with shatter coefficient $\mathcal{S}(\mathcal{F}, n)$. The Vapnik–Chervonenkis inequality also applies to weighted samples with slight modification.

Lemma S3.1 (VC inequality for weighted sample)**.** *Given a positive weighting function $w : \mathcal{X} \times \mathcal{Y} \to \mathbb{R}^+$ with $\|w\|_\infty < \infty$ and i.i.d. sample $(X_1, Y_1), \ldots, (X_n, Y_n)$, we have*

$$\mathbb{P}\left(\sup_{f\in\mathcal{F}} \left|\hat{R}_n(f) - R(f)\right| > \varepsilon\right) \leq 8\mathcal{S}(\mathcal{F}, n) \exp\left(\frac{-n\varepsilon^2}{32\|w\|_\infty^2}\right)$$

for all $n \geq 2\|w\|_\infty^2/\varepsilon^2$, where

- $\hat{R}_n(f) = \frac{1}{n}\sum_{i=1}^n w(X_i, Y_i)\mathbb{1}_{\{f(X_i)\neq Y_i\}}$,
- $R(f) = \mathbb{E}\left(w(X_1, Y_1)\mathbb{1}_{\{f(X_1)\neq Y_1\}}\right)$.

Proof. We modified the classic proof (see e.g., Devroye et al., 1996, Theorem 12.5 and 12.6) to obtain our weighted version. It suffices to notice that the shatter coefficient also dominates the maximum number of possible weighted classification outcome. For arbitrary sequences $(x_i, y_i, w_i = w(x_i, y_i))$, $i = 1, \ldots, n$, we write

$$\begin{aligned} N_{\mathcal{F}} &= \{(f(x_1), \ldots, f(x_n)) \in \{0,1\}^n : f \in \mathcal{F}\}, \\ I_{\mathcal{F}} &= \left\{\left(\mathbb{1}_{\{f(x_1\neq y_1)\}}, \ldots, \mathbb{1}_{\{f(x_n\neq y_n)\}}\right) : f \in \mathcal{F}\right\}, \\ I_{\mathcal{F},w} &= \left\{\left(w_1\mathbb{1}_{\{f(x_1\neq y_1)\}}, \ldots, w_n\mathbb{1}_{\{f(x_n\neq y_n)\}}\right) : f \in \mathcal{F}\right\}, \end{aligned}$$

so that shatter coefficient is defined as

$$\mathcal{S}(\mathcal{F}, n) := \max_{x_1,\ldots,x_n\in\mathcal{X}} |N_{\mathcal{F}}|,$$

where $|\cdot|$ is the number of elements in a set. Notice that $|I_{\mathcal{F},w}| \leq |I_{\mathcal{F}}| \leq |N_{\mathcal{F}}| \leq \mathcal{S}(\mathcal{F}, n)$, indeed since $\left(w_i\mathbb{1}_{\{f(x_i\neq y_i)\}}\right)_{i=1}^n \neq \left(w_i\mathbb{1}_{\{f'(x_i\neq y_i)\}}\right)_{i=1}^n$ implies $\left(\mathbb{1}_{\{f(x_i\neq y_i)\}}\right)_{i=1}^n \neq \left(\mathbb{1}_{\{f'(x_i\neq y_i)\}}\right)_{i=1}^n$ and subsequently $(f(x_i))_{i=1}^n \neq (f'(x_i))_{i=1}^n$ for some $f, f' \in \mathcal{F}$. Therefore $\mathcal{S}(\mathcal{F}, n) \geq \max_{x_1,\ldots,x_n\in\mathcal{X}} |I_{\mathcal{F},w}|$.

Follow the classic proof, we symmetrize first. Let $D_n' = \{(X_1', Y_1') : i = 1, \ldots, n\}$ be another set of i.i.d. sample identically distributed but independent to the original sample D_n, and write the associated empirical risk as

$$\hat{R}_n'(f) = \frac{1}{n}\sum_{i=1}^n \mathbb{1}_{\{f(X_i')\neq Y_i'\}}.$$

Write $\tilde{f} = \tilde{f}(D_n) \in \mathcal{F}$ such that $\left|\hat{R}_n(\tilde{f}) - R(\tilde{f})\right| > \sup_{f\in\mathcal{F}} \left|\hat{R}_n(f) - R(f)\right| - \delta$ for arbitrary $\delta > 0$. Then

$$\begin{aligned} &\mathbb{P}\left(\sup_{f\in\mathcal{F}} \left|\hat{R}_n(f) - \hat{R}_n'(f)\right| > \varepsilon/2\right) \\ &\geq \mathbb{P}\left(\left|\hat{R}_n(\tilde{f}) - \hat{R}_n'(\tilde{f})\right| > \varepsilon/2\right) \\ &\geq \mathbb{P}\left(\left|\hat{R}_n(\tilde{f}) - R(\tilde{f})\right| > \varepsilon \text{ and } \left|\hat{R}_n'(\tilde{f}) - R(\tilde{f})\right| < \varepsilon/2\right) \\ &= \mathbb{E}\left(\mathbb{1}_{\{|\hat{R}_n(\tilde{f})-R(\tilde{f})|>\varepsilon\}}\mathbb{P}\left(\left|\hat{R}_n'(\tilde{f}) - R(\tilde{f})\right| < \varepsilon/2|D_n\right)\right). \end{aligned} \tag{S3.2}$$

Denoting $U_i = w(X_i', Y_i')\mathbb{1}_{\{\tilde{f}(X_i')\neq Y_i'\}} - \mathbb{E}\left(w(X_i', Y_i')\mathbb{1}_{\{\tilde{f}(X_i')\neq Y_i'\}}|D_n\right)$, we can write

$$\hat{R}_n'(\tilde{f}) - R(\tilde{f}) = \frac{1}{n}\sum_{i=1}^n U_i.$$

Thus Chebyshev's inequality implies

$$\begin{aligned}
&\mathbb{P}\left(\left|\hat{R}_n'(\tilde{f}) - R(\tilde{f})\right| < \varepsilon/2|D_n\right)\\
&= \mathbb{P}\left(\left|\sum_{i=1}^n U_i\right| < \frac{n\varepsilon}{2}|D_n\right)\\
&\geq 1 - \frac{4}{n^2\varepsilon^2} n \operatorname{var}(U_i|D_n)\\
&\geq 1 - \frac{\|w\|_\infty^2}{n\varepsilon^2},
\end{aligned}$$

which is greater than 1/2 since we assumed $n \geq 2\|w\|_\infty^2/\varepsilon^2$. Plugging-in to (S3.2) gives

$$\mathbb{P}\left(\sup_{f\in\mathcal{F}}\left|\hat{R}_n(f) - R(f)\right| > \varepsilon\right) \leq 2\mathbb{P}\left(\sup_{f\in\mathcal{F}}\left|\hat{R}_n(f) - \hat{R}_n'(f)\right| > \varepsilon/2\right). \tag{S3.3}$$

Indeed, since

$$\mathbb{P}\left(\sup_{f\in\mathcal{F}}\left|\hat{R}_n(f) - R(f)\right| > \varepsilon\right) \leq \mathbb{P}\left(\left|\hat{R}_n(\tilde{f}) - R(\tilde{f})\right| > \varepsilon\right)$$

by definition of $\tilde{f}$ and that δ can be arbitrarily small.

Then let $\sigma_1, \ldots, \sigma_n$ be i.i.d. Rademacher random variables, further symmetrization implies

$$\begin{aligned}
&\mathbb{P}\left(\sup_{f\in\mathcal{F}}\left|\hat{R}_n(f) - \hat{R}_n'(f)\right| > \varepsilon/2\right)\\
&= \mathbb{P}\left(\sup_{f\in\mathcal{F}}\frac{1}{n}\left|\sum_{i=1}^n\left(w_i\mathbb{1}_{\{f(X_i)\neq Y_i\}} - w_i'\mathbb{1}_{\{f(X_i')\neq Y_i'\}}\right)\right| > \varepsilon/2\right)\\
&= \mathbb{P}\left(\sup_{f\in\mathcal{F}}\frac{1}{n}\left|\sum_{i=1}^n\sigma_i\left(w_i\mathbb{1}_{\{f(X_i)\neq Y_i\}} - w_i'\mathbb{1}_{\{f(X_i')\neq Y_i'\}}\right)\right| > \varepsilon/2\right)\\
&\leq \mathbb{P}\left(\sup_{f\in\mathcal{F}}\frac{1}{n}\left|\sum_{i=1}^n\sigma_i\left(w_i\mathbb{1}_{\{f(X_i)\neq Y_i\}}\right)\right| > \varepsilon/4 \text{ or } \sup_{f\in\mathcal{F}}\frac{1}{n}\left|\sum_{i=1}^n\sigma_i\left(w_i'\mathbb{1}_{\{f(X_i')\neq Y_i'\}}\right)\right| > \varepsilon/4\right)\\
&\leq 2\mathbb{P}\left(\sup_{f\in\mathcal{F}}\frac{1}{n}\left|\sum_{i=1}^n\sigma_i\left(w_i\mathbb{1}_{\{f(X_i)\neq Y_i\}}\right)\right| > \varepsilon/4\right)
\end{aligned} \tag{S3.4}$$

where $w_i = w(X_i, Y_i)$ and $w_i' = w(X_i', Y_i')$. The last inequality is due to that X_i, Y_i and X_i', Y_i' are identically distributed.

Next, recall that $|I_{\mathcal{F},w}| \le |I_{\mathcal{F}}| \le \mathcal{S}(\mathcal{F}, n)$ for any given data D_n. Therefore with $A_{i,f} = \sigma_i w_i \mathbb{1}_{\{f(X_i) \ne Y_i\}}$, we have

$$\begin{aligned}
\mathbb{P}\left(\sup_{f\in\mathcal{F}} \frac{1}{n}\left|\sum_{i=1}^n A_{i,f}\right| > \varepsilon/4\right) &= \mathbb{E}\left(\mathbb{P}\left(\sup_{f\in\mathcal{F}} \frac{1}{n}\left|\sum_{i=1}^n A_{i,f}\right| > \varepsilon/4 \big| D_n\right)\right) \\
&= \mathbb{E}\left(\mathbb{P}\left(\max_{f\in\mathcal{F}_{D_n}} \frac{1}{n}\left|\sum_{i=1}^n A_{i,f}\right| > \varepsilon/4 \big| D_n\right)\right) \\
&\le \mathbb{E}\left(\sum_{f\in\mathcal{F}_{D_n}} \mathbb{P}\left(\frac{1}{n}\left|\sum_{i=1}^n A_{i,f}\right| > \varepsilon/4 \big| D_n\right)\right) \\
&\le \mathbb{E}\left(|\mathcal{F}_{D_n}| \max_{f\in\mathcal{F}_{D_n}} \mathbb{P}\left(\frac{1}{n}\left|\sum_{i=1}^n A_{i,f}\right| > \varepsilon/4 \big| D_n\right)\right) \\
&\le \mathcal{S}(\mathcal{F}, n)\mathbb{E}\left(\sup_{f\in\mathcal{F}} \mathbb{P}\left(\frac{1}{n}\left|\sum_{i=1}^n A_{i,f}\right| > \varepsilon/4 \big| D_n\right)\right).
\end{aligned}$$

Here $\mathcal{F}_{D_n}$ is the smallest subset of $\mathcal{F}$ that spans $N_{\mathcal{F}}$ so that $N_{\mathcal{F}}$ also equals to $\{(f(X_1), \dots, f(X_n)) : f \in \mathcal{F}_{D_n}\}$. By definition of the shatter coefficient, $|\mathcal{F}_{D_n}| \le \mathcal{S}(\mathcal{F}, n)$. Further, use the previous argument for $|I_{\mathcal{F},w}| \le |I_{\mathcal{F}}| \le |N_{\mathcal{F}}|$, we can interchange the $\sup_{f\in\mathcal{F}}$ and $\max_{f\in\mathcal{F}_{D_n}}$ in the previous inequalities.

Further, the introduction of the Rademacher r.v. makes $A_{i,f}$ zero mean, so that by Hoeffding's inequality

$$\mathbb{P}\left(\frac{1}{n}\left|\sum_{i=1}^n A_{i,f}\right| > \varepsilon/4 \big| D_n\right) \le 2\exp\left(-\frac{n\varepsilon^2}{32\|w\|_\infty^2}\right)$$

for any $D_n \subset (\mathcal{X} \times \mathcal{Y})^n$ and $f \in \mathcal{F}$. Combined with (S3.4), we have

$$\mathbb{P}\left(\sup_{f\in\mathcal{F}} \left|\hat{R}_n(f) - \hat{R}'_n(f)\right| > \varepsilon/2\right) \le 4\mathcal{S}(\mathcal{F}, n)\exp\left(-\frac{n\varepsilon^2}{32\|w\|_\infty^2}\right) \tag{S3.5}$$

The desired inequality then follows combining (S3.3) and (S3.5) □

Corollary S3.1. *Under the conditions of Lemma S3.1, further if the Vapnik-–Chervonenkis dimension of class $\mathcal{F}$ satisfies $V_{\mathcal{F}} < \infty$, then for any $\delta \in (0, 1)$, with probability greater than $1 - \delta$, we have*

$$\sup_{f\in\mathcal{F}} \left|\hat{R}_n(f) - R(f)\right| \le 4\|w\|_\infty \sqrt{\frac{2}{n}\left(V_{\mathcal{F}} \log \frac{en}{V_{\mathcal{F}}} - \log\delta\right)}$$

for all $n > 2V_{\mathcal{F}}$.

Proof. Use Lemma S3.1 and Sauer's lemma (e.g., Devroye et al., 1996, theorem 13.3). □

S3.3 Justifying Weights on the Single Index

We provide some justification for adopting w_V. Intuitively, as Y is independent on X conditioning on $V = {\theta_0}^T X$, it suffices to discard X and work with V directly, as long as θ_0 is known. Assume that w_X and w_V are well-defined (see conditions in Section S4), recall we defined

$$w_{Y|X} = \mathbb{P}(Y = y|X = x, S = 0) / \mathbb{P}(Y = y|X = x, S = 1)$$

and $w_Y = w_{Y|V}$ as in (9), we have that $w_{Y|X}(x,y) = w_{Y|V}({\theta_0}^T x, y)$. (Again, $w_{Y|V}$ is used only here to distinguish from $w_{Y|X}$. In the main text and other sections of the Supplement, we use w_Y for $w_{Y|V}$ for simpler notation.) Further, since $V = {\theta_0}^T X$, we have that

$$\mathbb{E}[Y \mid X, S] = \varphi\left(\Delta_0(1-S) + {\theta_0}^T X\right) = \mathbb{E}[Y \mid V, S]$$

which implies

$$\mathbb{E}[g({\theta_0}^T X, Y) \mid X, S] = \mathbb{E}[Y \mid X, S]\left(g({\theta_0}^T X, 1) - g({\theta_0}^T X, 0)\right) + g({\theta_0}^T X, 0) = \mathbb{E}[g(V, Y) \mid V, S] \tag{S3.6}$$

for any measurable function g. In other words, $\mathbb{E}[g({\theta_0}^T X, Y) \mid X, S]$ is a function of (V, S). In particular,

$$\begin{aligned} &\mathbb{E}[g({\theta_0}^T X, Y) \mid X = x, S = 0] = \mathbb{E}[w_{Y|X}(X,Y) g({\theta_0}^T X, Y) \mid X = x, S = 1] \\ &= \mathbb{E}[w_{Y|V}(V,Y) g(V,Y) \mid V = {\theta_0}^T x, S = 1] = \mathbb{E}[g(V,Y) \mid V = {\theta_0}^T x, S = 0]. \end{aligned}$$

Similarly

$$\mathbb{E}[w_X(X) g({\theta_0}^T X) \mid S = 1] = \mathbb{E}[g({\theta_0}^T X) \mid S = 0] = \mathbb{E}[g(V) \mid S = 0] = \mathbb{E}[w_V(V) g(V) \mid S = 1] \tag{S3.7}$$

for any measurable function g of V. In sum, it suffices to tackle the data shift in V. For example, to re-write (5) as (10), first by (S3.6) that

$$\begin{aligned} &\mathbb{E}[\mathbb{1}_{\left\{\varphi\left(\Delta_0 + {\theta_0}^T X\right) \geq c\right\}} (1-Y) w_{Y|X}(X,Y) \mid X, S = 1] \\ &= \mathbb{E}[\mathbb{1}_{\left\{\varphi\left(\Delta_0 + {\theta_0}^T X\right) \geq c\right\}} (1-Y) w_{Y|V}({\theta_0}^T X, Y) \mid X, S = 1] \\ &= \mathbb{E}[\mathbb{1}_{\{\varphi(\Delta_0 + V) \geq c\}} (1-Y) w_{Y|V}(V,Y) \mid V, S = 1], \end{aligned}$$

which is function of $V = {\theta_0}^T X$; thus

$$\begin{aligned} \text{RHS of (5)} &= \mathbb{E}[\mathbb{1}_{\{\eta(X) \geq c\}} (1-Y) w_{Y|X}(X,Y) w_X(X) \mid S = 1] \\ &= \mathbb{E}[\mathbb{1}_{\left\{\varphi\left(\Delta_0 + {\theta_0}^T X\right) \geq c\right\}} (1-Y) w_{Y|X}(X,Y) w_X(X) \mid S = 1] \\ &= \mathbb{E}[w_X(X)\, \mathbb{E}[\mathbb{1}_{\left\{\varphi\left(\Delta_0 + {\theta_0}^T X\right) \geq c\right\}} (1-Y) w_{Y|X}(X,Y) \mid X, S = 1] \mid S = 1] \\ &= \mathbb{E}[w_V(V)\, \mathbb{E}[\mathbb{1}_{\{\varphi(\Delta_0 + V) \geq c\}} (1-Y) w_{Y|V}(V,Y) \mid V, S = 1] \mid S = 1] \\ &= \mathbb{E}[\mathbb{1}_{\{\varphi(\Delta_0 + V) \geq c\}} (1-Y) w_V(V) \cdot w_{Y|V}(V,Y) \mid S = 1] \\ &= \text{RHS of (10)}, \end{aligned}$$

where the 4th equation by (S3.7).

It is worth noting that while $x \mapsto w_X$ and $x \mapsto w_V$ do not take the same value but can be connected by change of variable, e.g., taking $u_j = \sum_{i \leq j} \theta_{0,i} x_i$, then

$$\begin{aligned} &w_V(v) f_{V,\text{sr}}(v) = f_{V,\text{tg}}(v) = \int f_{X,\text{tg}}\left(\frac{u_1}{\theta_{0,1}}, \ldots, \frac{u_{d-1} - u_{d-2}}{\theta_{0,d-1}}, \frac{v - u_{d-1}}{\theta_{0,d}}\right) \frac{du_1 \cdots du_{d-1}}{\theta_{0,1} \cdots \theta_{0,d}} \\ &= \int (w_X \cdot f_{X,\text{sr}})\left(\frac{u_1}{\theta_{0,1}}, \ldots, \frac{u_{d-1} - u_{d-2}}{\theta_{0,d-1}}, \frac{v - u_{d-1}}{\theta_{0,d}}\right) \frac{du_1 \cdots du_{d-1}}{\theta_{0,1} \cdots \theta_{0,d}}. \end{aligned}$$

S4 List of Assumptions

(A1) Θ is a compact neighborhood of θ_0 where all elements in θ are non-zero for all $\theta \in \Theta$. Also θ_0 is the unique in Θ satisfying (6).

(A2) $f_{X,\mathrm{sr}}$ and $f_{X,\mathrm{tg}}$ are compactly supported and three times continuously differentiable with Lipschitz continuous third derivatives. Also $\mathcal{X}_{\mathrm{tg}} = \{x : f_{X,\mathrm{tg}}(x) > 0\} \subset \mathcal{X}$.

(A3) The mapping $x \mapsto \mathbb{E}[Y^{\mathrm{sr}} \mid X^{\mathrm{sr}} = x]$ is three times continuously differentiable with Lipschitz continuous third derivatives and bounded away from zero and one on $\mathcal{X}$. φ is strictly monotone.

(A4) The mapping $x \mapsto \mathbb{P}(S = 1|X = x)$ is twice continuously differentiable and bounded away from zero and one on $\mathcal{X}$.

(A5) The baseline shift of interest is bounded: $\underline{\Delta_0} \leq \Delta_0 \leq \overline{\Delta_0}$. The single index of the target population is bounded in the way that: given any $\theta \in \Theta$, the set $\{\Delta + \theta^T x : \underline{\Delta_0} \leq \Delta \leq \overline{\Delta_0}, x \in \operatorname{supp} f_{X,\mathrm{tg}}\}$ is contained in $\mathcal{V}_\theta$.

(P1) There exists a unique c^*_α such that $\mathbb{P}(\eta(X^{\mathrm{tg}}) \geq c^*_\alpha | Y^{\mathrm{tg}} = 0) = \alpha$.

(P2) (margin assumption) There exist positive constants C_0 and $\bar{\gamma}$, such that for all $\delta \geq 0$,

$$\mathbb{P}\left(\left|\eta(X^{\mathrm{tg}}) - c^*_\alpha\right| \leq \delta | Y^{\mathrm{tg}} = 0\right) \leq C_0 \delta^{\bar{\gamma}}.$$

(P3) (level α detection) There exist positive constants C_1 and $\underline{\gamma}$, such that for any δ in a small right neighborhood of 0,

$$\mathbb{P}\left(c^*_\alpha - \delta \leq \eta(X^{\mathrm{tg}}) \leq c^*_\alpha | Y^{\mathrm{tg}} = 0\right) \wedge \mathbb{P}\left(c^*_\alpha \leq \eta(X^{\mathrm{tg}}) \leq c^*_\alpha + \delta | Y^{\mathrm{tg}} = 0\right) \geq C_1 \delta^{\underline{\gamma}}.$$

We impose those assumptions so that the density ratio and $Y|X$ are well-behaved. In short, conditions (A1) and (A2) ensure the density ratio $f_{X,\mathrm{tg}}/f_{X,\mathrm{sr}}$ and $f_{V,\mathrm{tg}}/f_{V,\mathrm{sr}}$ are well-defined and bounded on $\mathcal{X}$ for $\theta \in \Theta$, while adding (A4) making its consistent estimation possible. The compact-support condition in (A2) is mainly technical. More fundamentally, the theory requires adequate overlap between the source and target populations so that these density ratios remain well defined on the target support. Condition (A3) provides smoothness for the mapping $(v, \theta) \mapsto \mathbb{E}[Y^{\mathrm{sr}} \mid \theta^T X^{\mathrm{sr}} = v]$ for $\theta \in \Theta$ by change of variable, as well as the existence and uniqueness of $\hat{\Delta}_0$ in (14). Condition (A5) ensures w_Y is well-defined as in (9), where $(\underline{\Delta_0}, \overline{\Delta_0})$ is effectively the wiggling room for $\hat{\Delta}_0$. Also it ensures for sufficiently large sample and consistent $\hat{\Delta}_0$ and $\hat{\theta}$, we have $\hat{\Delta}_0 + \hat{\theta}^T x \in \mathcal{V}$ for $x \in \mathcal{X}_{\mathrm{tg}}$, and $\hat{w}_V\left(\hat{\theta}^T x\right) = o_p(1)$ when $x \in \mathcal{X} - \mathcal{X}_{\mathrm{tg}}$.

We assume the following for consistency of kernel smoothing. Note that (K1) is slightly stronger than necessary for Lemma S6.1 due to Proposition S7.1.

(K1) K is non-negative, supported on $[-1, 1]$. It is twice continuously differentiable with Lipschitz continuous second derivative. $\int K(u)du = 1$, $\int uK(u)du = 0$.

In addition, Proposition 3.1 assumes the bandwidths h_φ and h_ψ in (12) and (13) shrink to zero as $n \to \infty$ following

(B1) $\max(h_\varphi, h_\psi) \to 0$, $nh_\psi^2 \to \infty$, $\log n/(nh_\psi) \to 0$, $|\log h_\varphi|/(nh_\varphi^4) \to 0$, and $nh_\varphi^8 \to 0$.

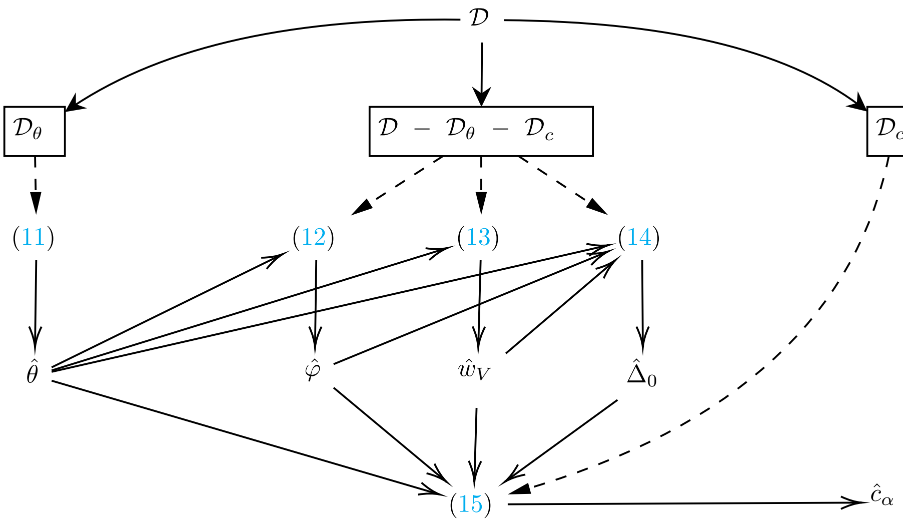

Figure S4.1: Data split to untangle dependency. The sample $\mathcal{D}$ is split into three parts: the $\mathcal{D}_{\theta_0}$ for θ_0, the $\mathcal{D}_c$ for c, and the rest for other components. A dotted line indicates the estimation formula is evaluated on the specific partition of the data.

S4.1 Data Split and Growth in Sizes

For simpler derivation, we split the data by assuming the following conditions [D1]–[D3] throughout the theoretical discussion unless otherwise specified. Dependency among components, e.g., $\hat\eta$, $\hat w$, X^{sr}, and Y^{sr} in (15), renders analysis of excess empirical risk difficult. Data splitting is a common approach to simplify theoretical treatments of plug-in NP classifiers (e.g., Tong, 2013). Some parts of the data are dedicated to estimating certain quantities while others are used nowhere else, so dependency is untangled via conditioning when necessary. For theoretical purposes only, assume

[D1] $\mathcal{D}_\theta = \{(X_i^{\mathrm{sr}}, Y_i^{\mathrm{sr}}) : i \in \mathcal{I}_\theta\}$ is dedicated to computing $\hat\theta$ via (11).

[D2] $\mathcal{D}_c = \{(X_i^{\mathrm{sr}}, Y_i^{\mathrm{sr}}) : Y_i^{\mathrm{sr}} = 0, i \in \mathcal{I}_c\}$ is dedicated to computing $\hat c_\alpha$ via (15).

Here, $\mathcal{I}_\theta$ and $\mathcal{I}_c$ are subsets of the subject index $\mathcal{I} = \{1, \ldots, n_{\mathrm{sr}} + n_{\mathrm{tg}}\}$. That is, use $\mathcal{D}_\theta$ first to produce $\hat\theta$, then other quantities $\hat\varphi$, $\hat w_V$, and $\hat\Delta_0$ are computed based on $\hat\theta$ and $\mathcal{D} - \mathcal{D}_\theta - \mathcal{D}_c$. In the end, $\hat c_\alpha$ is obtained based on the previous estimates and sample $\mathcal{D}_c$. Figure S4.1 provides a simple visualization. Hence we have a sum of i.i.d. random variables in (12) conditioning on $\mathcal{D}_\theta$; and in (15) conditioning on $\mathcal{D} - \mathcal{D}_c$. Note that $\mathcal{D}_c$ only needs to contain negative $Y = 0$ when tackling problem (1).

We further assume the sizes of the split data subsets are asymptotically equivalent so that it suffices to write $n \to \infty$ with $n = |\mathcal{D}|$ for the increasing sizes of all $\mathcal{D}$, $\mathcal{D}_\theta$, and $\mathcal{D}_c$. More precisely, assume $0 < \mathbb{P}(S = 1) < 1$ so that the size of $\mathcal{D}_{\mathrm{sr}}$ and $\mathcal{D}_{\mathrm{tg}}$ both diverge as $n \to \infty$. Further, assume the sizes of the dedicated $\mathcal{D}_\theta$ and $\mathcal{D}_c$ are proportional to n, so that

[D3] $n \asymp n_{\mathrm{sr}} \asymp n_{\mathrm{tg}} \asymp n_\theta \asymp n_\varphi \asymp n_{w_V} \asymp n_\Delta \asymp n_c$ as $n \to \infty$,

where $n_\theta = |\mathcal{D}_\theta|$, $n_\varphi = n_{\mathrm{sr}} - n_\theta - n$, $n_{w_V} = n_{\mathrm{tg}} + n_{\mathrm{sr}} - n_\theta - n$, $n_\Delta = n_{\mathrm{sr}} - n_\theta - n$, and $n_c = |\mathcal{D}_c|$ are the sizes of samples utilized for estimating θ_0, φ, w_V, Δ_0, and c_α^* via (11), (12), (13), (14), and (15), respectively. Here "$\asymp$" means, e.g., $n = O(n_{\mathrm{sr}})$ and $n_{\mathrm{sr}} = O(n)$.

S5 Consistency of True/False Positive Rate

We now establish the optimality of the proposed rule given any consistent estimators of w_X, w_Y, and τ. Denote $\mathcal{X} = \{x : f_{X,\mathrm{sr}}(x) > 0\} \subset \mathbb{R}^d$ as the support for $f_{X,\mathrm{sr}}$ and $\mathcal{V} = \bigcup_{\theta\in\Theta} \mathcal{V}_\theta$ with $\mathcal{V}_\theta = \left\{\theta^T x : x \in \mathcal{X}\right\}$ as the domain for single index. Write w, w_V, and w_Y for $v = v(x;\theta) = \theta^T x$, $x \in \mathcal{X}$, and $y = 0, 1$ with

$$w_V(v) = w_V(v;\theta) = \frac{f_{V,\mathrm{tg},y}(v;\theta)}{f_{V,\mathrm{sr},y}(v;\theta)}, \quad w_Y(v,y) = w_Y(v,y;\theta) = \frac{\mathbb{P}\left(Y^{\mathrm{tg}} = y|\theta^T X^{\mathrm{tg}} = v\right)}{\mathbb{P}\left(Y^{\mathrm{sr}} = y|\theta^T X^{\mathrm{sr}} = v\right)},$$

and $w_\theta(v,y) = w(v,y;\theta) = w_V(v;\theta)w_Y(v,y;\theta)$; where $f_{V,\mathrm{tg},y}(v;\theta)$ is the density of $v = \theta^T X^{\mathrm{tg}}$ conditioning on $Y^{\mathrm{tg}} = y$, similarly $f_{V,\mathrm{sr},y}(v;\theta)$ is the same conditional density for the source population. We write $\hat{w}_V(\cdot;\theta), \hat{w}_Y(\cdot;\theta)$, and $\hat{w}(\cdot;\theta)$ as their estimate based on $\theta^T X_i^{\mathrm{sr}}$, $\theta^T X_j^{\mathrm{tg}}$, and the selection variable S_i.

To describe the consistency of plugged-in components, we first assume and later prove that the given estimator $\hat{\theta} = \hat{\theta}_n$ satisfies

(R1) $\|\hat{\theta} - \theta_0\|_\infty = O_p\left(a_n^{(\theta)}\right)$,

and that the sample weights and the conditional risk are consistently estimated such that

(R2) $\sup_{x\in\mathcal{X},\theta\in\Theta} \left|\hat{w}_V(\theta^T x;\theta) - w_V(\theta^T x;\theta)\right| = O_p\left(a_n^{(w,X)}\right)$;

(R3) $\sup_{x\in\mathcal{X}_{\mathrm{tg}},y\in\{0,1\},\theta\in\Theta_n} \left|\hat{w}_Y(\theta^T x,y;\theta) - w_Y(\theta^T x,y;\theta)\right| = O_p\left(a_n^{(w,Y)}\right)$;

(R4) $\sup_{x\in\mathcal{X}} |\hat{\tau}(x) - \tau(x)| = O_p\left(a_n^{(\tau)}\right)$;

(R5) $\sup_{x\in\mathcal{X}_{\mathrm{tg}},y\in\{0,1\}} \left|\hat{w}_Y(\hat{\theta}^T x,y;\hat{\theta}) - w_Y({\theta_0}^T x,y;\theta_0)\right| = O_p\left(a_n^{(\Delta)}\right)$;

where Θ_n is some sequence of shrinking compact neighborhoods of θ_0 with $\cap_n\Theta_n = \theta_0$; while $a_n^{(\theta)}$, $a_n^{(w,X)}$, $a_n^{(w,Y)}$, $a_n^{(\Delta)}$, and $a_n^{(\tau)}$ are some diminishing positive sequences as $n \to \infty$. Conditions (R2) and (R3) ensures the weighted $\left(\theta^T X^{\mathrm{sr}}, Y^{\mathrm{sr}}\right)$-distribution converges to that of $\left(\theta^T X^{\mathrm{tg}}, Y^{\mathrm{tg}}\right)$ uniformly over θ on some neighborhood of θ_0. Condition (R4) and (R5) ensures the conditional risk in the target population is consistently estimated even with $\hat{\theta}$, so is the $Y|X$-ratio. The O_p-rates in (R2) and (R3) need to be "uniform" over some θ. Unlike (R2), we cannot take supremum over $\theta \in \Theta$ in (R3) under model (6), since it is only at $\theta = \theta_0$ yet not necessarily at other θ that we assume $\mathbb{E}[Y^{\mathrm{tg}} \mid \theta^T X^{\mathrm{tg}} = v] = \varphi(\Delta_0 + v)$. To properly utilize the form of baseline shift, we need a consistent $\hat{\theta}$. Thus the consistency of $\hat{w}_Y$ is only achievable in a shrinking Θ_n, as shown in the proof of Theorem 3.1.

For simpler notation, we assert the following.

Proposition S5.1. *Under (A1), (A2), (A3), (A4), and (A5), conditions (R1), (R2) and (R3) imply*

(R6) $\sup_{x\in\mathcal{X}_{\mathrm{tg}},y\in\{0,1\}} \left|\hat{w}\left(\hat{\theta}^T x,y;\hat{\theta}\right) - w\left(\hat{\theta}^T x,y;\hat{\theta}\right)\right| = O_p(a_n^{(w)})$;

conditions (R4) and (R5) imply

(R7) $\sup_{\mathcal{X}_{\mathrm{tg}}} |\hat{\eta}(x) - \eta(x)| = O_p(a_n^{(\eta)})$;

where $a_n^{(w)} = a_n^{(w,X)} + a_n^{(w,Y)}$ *and* $a_n^{(\eta)} = a_n^{(\tau)} + a_n^{(\Delta)}$.

Proof. (R7) is obvious. For (R6), note that there exists $\varepsilon, M > 0$ so that

$$\begin{aligned}
&\mathbb{P}\left(\left|\hat{w}_Y\left(\hat{\theta}^T x, y; \hat{\theta}\right) - w_Y\left(\hat{\theta}^T x, y; \hat{\theta}\right)\right| > M a_n^{(w,Y)}\right) \\
\leq\, &\mathbb{P}\left(\hat{\theta} \notin \Theta_n\right) + \mathbb{P}\left(\sup_{\theta \in \Theta_n}\left|\hat{w}_Y\left(\theta^T x, y; \theta\right) - w_Y\left(\theta^T x, y; \theta\right)\right| > M a_n^{(w,Y)}\right) \\
<\, &\varepsilon
\end{aligned}$$

for sufficiently large n_θ and n. This is a uniform rate for any consistent $\hat{\theta}$.

□

A side note is that $\hat{w}$ also approximate w well even for $x \in \mathcal{X} - \mathcal{X}_{\mathrm{tg}}$. Since

$$\begin{aligned}
&\left|\hat{w}\left(\theta^T x, y; \theta\right) - w\left(\theta^T x, y; \theta\right)\right| \\
=\, &\left|\hat{w}_V\left(\theta^T x\right)\hat{w}_Y\left(\theta^T x, y\right) - w_V\left(\theta^T x\right) w_Y\left(\theta^T x, y\right)\right| \\
\lesssim\, &\sup\left\{\left|\hat{w}_V\left(\theta^T x\right)\right| : x \in \mathcal{X} - \mathcal{X}_{\mathrm{tg}}, \theta \in \Theta\right\} \\
&+ \sup_{x \in \mathcal{X}_{\mathrm{tg}}, \theta}\left|\hat{w}_V\left(\theta^T x\right)\right| \sup_{x \in \mathcal{X}_{\mathrm{tg}}, y, \theta}\left|\hat{w}_Y\left(\theta^T x, y\right) - w_Y\left(\theta^T x, y\right)\right| \\
&+ \sup_{x \in \mathcal{X}_{\mathrm{tg}}, \theta}\left|w_Y\left(\theta^T x, y\right)\right| \sup_{x \in \mathcal{X}_{\mathrm{tg}}, y, \theta}\left|\hat{w}_X\left(\theta^T x\right) - w_X\left(\theta^T x\right)\right|,
\end{aligned}$$

the $\mathcal{X} - \mathcal{X}_{\mathrm{tg}}$ part in the covariate domains are eliminated by w_V.

Remark (Randomized rules)**.** When an exact equality in (5), (10), or (15) is unachievable, we resort to randomized rule by setting $R^*_{\mathrm{tg}}(X) = 1$ if $\eta(X) > c^*$, $R^*_{\mathrm{tg}}(X) = \gamma^*$ if $\eta(X) = c^*$, and 0 otherwise, so that the decision is assigned by random with positive probability equal to $R^*_{\mathrm{tg}}(X)$. Here $c^* = \inf\left\{c : \mathbb{E}[\mathbb{1}_{\{\eta(X)>c\}}(1-Y)w(X,Y) \mid S=1] \leq \alpha\mathbb{P}\left(Y=0|S=0\right)\right\}$ and γ^* is some constant such that $\alpha\mathbb{P}\left(Y=0|S=0\right) = \mathbb{E}[\left(\mathbb{1}_{\{\eta(x)>c\}} + \gamma^*\mathbb{1}_{\{\eta(x)=c\}}\right)(1-Y)w(X,Y) \mid S=1]$.

Especially in practice, the weighted empirical FPR is a step function of c. Therefore, randomized decision rules may be adopted with a $\hat{\gamma}$ such that

$$\frac{\sum_{i=1}^{n_{\mathrm{sr}}}\left(\hat{\gamma}\mathbb{1}_{\left\{\hat{\eta}\left(X_i^{\mathrm{sr}}\right)=\hat{c}_\alpha\right\}} + \mathbb{1}_{\left\{\hat{\eta}\left(X_i^{\mathrm{sr}}\right)>\hat{c}_\alpha\right\}}\right)\hat{w}_i\left(1-Y_i^{\mathrm{sr}}\right)}{\sum_{i=1}^{n_{\mathrm{sr}}}\hat{w}_i\left(1-Y_i^{\mathrm{sr}}\right)} = \alpha.$$

□

The following two propositions are derived from proposition 4 and theorem 3 in Tong (2013). Here $\hat{R}_{c,\gamma}(x) := \mathbb{1}_{\{\hat{\eta}(x)>c\}} + \gamma\mathbb{1}_{\{\hat{\eta}(x)=c\}}$.

Proposition S5.2. *Under (R6), we have*

$$\sup_{0\leq c,\gamma\leq 1}\left|\mathrm{FPR}\left(\hat{R}_{c,\gamma}\right) - \widehat{\mathrm{FPR}}\left(\hat{R}_{c,\gamma}\right)\right| = O_p\left(a_n^{(w)} + \sqrt{\log n_c/n_c}\right)$$

as $n_c \to \infty$ and $n_w \to \infty$.

Proof of Proposition S5.2. By definition of w,

$$\mathbb{E}\left(g(\theta^T X^{\mathrm{tg}})|Y^{\mathrm{tg}}=0\right) = \frac{p_{0,\mathrm{sr}}}{p_{0,\mathrm{tg}}}\int_{\mathcal{X}} g(t)w(t,0;\theta)f_{V,\mathrm{sr},0}(t;\theta)dt \tag{S5.1}$$

for any $\theta \in \Theta$ and measurable function g, where $p_{0,\text{sr}} = \mathbb{P}\left(Y^{\text{sr}} = 0\right)$ and $p_{0,\text{tg}} = \mathbb{P}\left(Y^{\text{tg}} = 0\right)$. Hence the false positive rate of the estimated rule (fixing $\hat{\eta}$ and $\hat{\theta}$ by conditioning on $\mathcal{D}_\eta := \mathcal{D} - \mathcal{D}_c$) is

$$\mathbb{P}\left(\hat{\eta}(X^{\text{tg}}) \geq c | Y^{\text{tg}} = 0, \mathcal{D}_\eta\right) = \frac{p_{0,\text{sr}}}{p_{0,\text{tg}}} \mathbb{E}\left(w(\hat{\theta}^T X^{\text{sr}}, Y^{\text{sr}}; \hat{\theta}) \mathbb{1}_{\{\hat{\eta}(X^{\text{sr}}) \geq c\}} | Y^{\text{sr}} = 0, \mathcal{D}_\eta\right).$$

Denote $\hat{w}_i = \hat{w}(\hat{\theta}^T X_i^{\text{sr}}, Y_i^{\text{sr}}; \hat{\theta})$, $w_i = w(\hat{\theta}^T X_i^{\text{sr}}, Y_i^{\text{sr}}; \hat{\theta})$, and after conditioning on $\mathcal{D}_\eta$ it suffices to consider

$$T_0 = \sup_c \left| \frac{\sum_{\mathcal{D}_c} \hat{w}_i \mathbb{1}_{\{\hat{\eta}(X_i) \geq c\}}}{\sum_{\mathcal{D}_c} \hat{w}_i} - \mathbb{P}\left(\hat{\eta}(X^{\text{tg}}) \geq c | Y^{\text{tg}} = 0, \mathcal{D}_\eta\right) \right|.$$

Our goal is to bound T_0. (Recall $\mathcal{D}_c$ contains only observations with $Y_i^{\text{sr}} = 0$.) Note that

$$\begin{aligned} T_0 &= \sup_c \left| \frac{1}{n_c} \sum_{\mathcal{D}_c} \hat{w}_i \mathbb{1}_{\{\hat{\eta}(X_i) \geq c\}} \frac{n_c}{\sum_{\mathcal{D}_c} \hat{w}_i} - \mathbb{P}\left(\hat{\eta}(X^{\text{tg}}) \geq c | Y^{\text{tg}} = 0, \mathcal{D}_\eta\right) \right| \\ &\leq \frac{p_{0,\text{sr}}}{p_{0,\text{tg}}} \sup_c \left| \frac{1}{n_c} \sum_{\mathcal{D}_c} w_i \mathbb{1}_{\{\hat{\eta}(X_i) \geq c\}} - \mathbb{E}\left(w(\hat{\theta}^T X^{\text{sr}}, Y^{\text{sr}}; \hat{\theta}) \mathbb{1}_{\{\hat{\eta}(X^{\text{sr}}) \geq c\}} | Y^{\text{sr}} = 0, \mathcal{D}_\eta\right) \right| \\ &\quad + \frac{1}{n_c} \left| \frac{n_c}{\sum_{\mathcal{D}_c} \hat{w}_i} - \frac{p_{0,\text{sr}}}{p_{0,\text{tg}}} \right| \sum_{\mathcal{D}_c} \hat{w}_i + \frac{p_{0,\text{sr}}}{p_{0,\text{tg}}} \max_i |\hat{w}_i - w_i|. \end{aligned} \tag{S5.2}$$

For simplicity, we write the three terms in the last inequality as T_1, T_2, and T_3. Clearly $T_3 = O_p\left(a_n^{(w)}\right)$ by (R6).

We claim that $T_1 = O_p(\sqrt{\log n_c / n_c})$ by VC inequality. Indeed, it suffices to note that the class of candidate rules $\left\{x \mapsto \mathbb{1}_{\{\hat{\eta}(x) \geq c\}} : 0 \leq c \leq 1\right\}$ is a VC class with VC dimension 1 and then apply Corollary S3.1 so that $\mathbb{P}\left(T_1 > t | \mathcal{D}_\eta\right) \leq \varepsilon$ with $t = M\sqrt{\log n_c / n_c}$ for all $n_c > N_\varepsilon$, where N_ε and $M > 0$ are constants determined by $p_{0,\text{sr}}$, $p_{0,\text{tg}}$, $\sup_\theta \|w_\theta\|_\infty$ and ε. The fact that the inequality holds regardless of $\mathcal{D}_\eta$ implies $\mathbb{P}\left(T_1 > t\right) = \mathbb{E}\left(\mathbb{P}\left(T_1 > t | \mathcal{D}_\eta\right)\right)$ vanishes at the same rate.

We tackle the terms T_2 utilizing the uniform convergence of $\hat{w}$. Note that

$$\begin{aligned} &\left| \frac{1}{n_c} \sum_{\mathcal{D}_c} \hat{w}_i - \mathbb{E}\left(w\left(\hat{\theta}^T X^{\text{sr}}, Y^{\text{sr}}; \hat{\theta}\right) | Y^{\text{sr}} = 0\right) \right| \\ &\leq \max_i |\hat{w}_i - w_i| + \left| \frac{1}{n_c} \sum_{\mathcal{D}_c} w_i - \mathbb{E}\left(w\left(\hat{\theta}^T X^{\text{sr}}, Y^{\text{sr}}; \hat{\theta}\right) | Y^{\text{sr}} = 0\right) \right|. \end{aligned}$$

Note that $\mathbb{E}\left(w\left(X^{\mathrm{sr}}, Y^{\mathrm{sr}}; \hat{\theta}\right)|Y^{\mathrm{sr}}=0\right) = p_{0,\mathrm{tg}}/p_{0,\mathrm{sr}}$ by (S5.1), so that

$$
\begin{aligned}
&\mathbb{P}\left(T_2 > t\right) \\
&\leq \mathbb{P}\left(\frac{1}{n_c}\sum_{\mathcal{D}_c}\hat{w}_i > B\right) + \mathbb{P}\left(\left|\frac{1}{n_c}\sum_{\mathcal{D}_c}\hat{w}_i - \frac{p_{0,\mathrm{tg}}}{p_{0,\mathrm{sr}}}\right| > \frac{t}{LB}\right) \\
&\leq \mathbb{P}\left(\frac{1}{n_c}\sum_{\mathcal{D}_c}\hat{w}_i > B\right) + \mathbb{P}\left(\max_i|\hat{w}_i - w_i| > \frac{t}{2LB}\right) \\
&\quad + \mathbb{P}\left(\left|\frac{1}{n_c}\sum_{\mathcal{D}_c}w_i - \frac{p_{0,\mathrm{tg}}}{p_{0,\mathrm{sr}}}\right| > \frac{t}{2LB}\right) + o(1),
\end{aligned}
$$

where B is some sufficiently large constant. L is the supremum of the absolute value of derivatives of $t \mapsto 1/t$ in a neighborhood of $t = p_{0,\mathrm{tg}}/p_{0,\mathrm{sr}}$ so that the probability that $\sum_{\mathcal{D}_c} w_i/n_c$ falls outside of such neighborhood is neglectable (that $o(1)$ in the last). $L < \infty$ since $p_{0,\mathrm{tg}}/p_{0,\mathrm{sr}}$ is bounded away from 0. The first term vanishes since (R6) implies $\sum_{\mathcal{D}_c}\hat{w}_i/n_c = O_p(1)$. The last term in the previous inequality can be bounded by central limit theorem. Therefore, take $t \asymp a_n^{(w)} + 1/\sqrt{n_c}$, the tail probability $\mathbb{P}\left(T_2 > t\right)$ can be arbitrarily small for sufficiently large $B > 0$ and n_c. Thus $T_2 = O_p(a_n^{(w)} + 1/\sqrt{n_c})$.

Finally, it suffices to combine the previous results in (S5.2) to obtain the desired rate for the non-randomized component $\mathbb{1}_{\{\hat{\eta}(t)>c\}}$. Similar arguments apply to the randomized component $\gamma\mathbb{1}_{\{\hat{\eta}(t)=c\}}$. Observe that

$$
\begin{aligned}
&\sup_{\gamma,c}\left|\sum_{\mathcal{D}_c}\gamma\hat{w}_i\mathbb{1}_{\{\hat{\eta}(X_i^{\mathrm{sr}})=c\}}/\sum_{\mathcal{D}_c}\hat{w}_i - \gamma\mathbb{P}\left(\hat{\eta}(X^{\mathrm{tg}}) = c|Y^{\mathrm{tg}}=0,\mathcal{D}_\eta\right)\right| \\
&\leq \frac{p_{0,\mathrm{sr}}}{p_{0,\mathrm{tg}}}\sup_c\left|\frac{1}{n_c}\sum_{\mathcal{D}_c}w_i\mathbb{1}_{\{\hat{\eta}(X_i^{\mathrm{sr}})=c\}} - \mathbb{E}\left(w\left(X^{\mathrm{sr}}, Y^{\mathrm{sr}}; \hat{\theta}\right)\mathbb{1}_{\{\hat{\eta}(X^{\mathrm{sr}})=c\}}|Y^{\mathrm{sr}}=0,\mathcal{D}_\eta\right)\right| \\
&\quad + T_2 + T_3 \\
&= O_p\left(a_n^{(w)} + \sqrt{\log n_c/n_c}\right),
\end{aligned}
$$

where T_2 and T_3 are those in (S5.2). Note that the VC dimension of class $\left\{\mathbb{1}_{\{\hat{\eta}(t)=c\}} : 0 \leq c \leq 1\right\}$ is 1. Condition (P1) also implies η is strictly monotone near $\{x : \eta(x) = c_\alpha^*\}$, therefore by definition, $\hat{\gamma} \lesssim_p a_n^{(w)} + \sqrt{\log n_c/n_c}$. □

Remark (Randomized rule continue)**.** It is not guaranteed that a cutoff $\hat{c}_\alpha$ exists for any given $\alpha \in (0,1)$ so that the infimum is achieved in (15) since the empirical risk is a step function. Though, Proposition S5.2 ensures that the empirical risk will not exceed the actual risk by large magnitude, hence the later is controlled with probability trending to 1. The primary reason that we employ randomized rule is to achieve such a exact false positive rate, so that $\mathrm{FPR}(\hat{R}_{\hat{c}_\alpha,\hat{\gamma}_\alpha}) - \alpha = O_p\left(a_n^{(w)} + \sqrt{\log n_c/n_c}\right)$.

The previous result bounds the empirical false positive rate for any decision function $\mathcal{X} \to \{0,1\}$. If further $\hat{\eta}$ consistently estimate η uniformly on $\mathcal{X}_{\mathrm{tg}} = \{x : f_{X,\mathrm{tg}}(x) > 0\}$, we can show the true positive rate is indeed maximized.

Proposition S5.3. *Under (A3), (P1), (P2), (P3), (R6), and (R7), we have*

$$\left|\mathrm{TPR}(\hat{R}) - \mathrm{TPR}^*\right| = O_p\left(\left(a_n^{(w)} + \sqrt{\frac{\log n_c}{n_c}}\right)^{\min\left\{1, \frac{1+\bar{\gamma}}{\underline{\gamma}}\right\}} + \left(a_n^{(\eta)}\right)^{1+\bar{\gamma}}\right)$$

as n_c, n_η, and $n_w \to \infty$, where TPR^* is the true positive rate for the optimal rule $\mathbb{1}_{\{\eta(x) \geq c_\alpha^*\}}$.

Proof of Proposition S5.3. Denote the true and estimated "rejection" domain as $G^* = \{\eta < c_\alpha^*\}$ and $\hat{G} = \{\hat{\eta} < \hat{c}_\alpha\}$. For simplicity of notations, we write P_0 (or P_1) as the conditional distribution of target covariates (X^{tg}) conditioning on $\mathcal{D}_\eta$, $\mathcal{D}_c$, and $Y^{\mathrm{tg}} = 0$ (or $Y^{\mathrm{tg}} = 1$). Observe that

$$\mathrm{TPR}(R^*) - \mathrm{TPR}(\hat{R}) = \mathbb{E}\left[P_1(\hat{G}) - P_1(G^*) \;\middle|\; Y^{\mathrm{tg}} = 1\right],$$

it suffices to tackle the difference in P_1. Write $f_{X,\mathrm{tg},0}, f_{X,\mathrm{tg},1}$ as the conditional density for P_0 and P_1 respectively. Note that $f_{X,\mathrm{tg},0}(x) \propto (1 - \eta(x))\, f_{X,\mathrm{tg}}(x)$, condition (A3) implies that $f_{X,\mathrm{tg},1}(x) = 0$ if $f_{X,\mathrm{tg},0}(x) = 0$, therefore we can write $dP_1 = f_{X,\mathrm{tg},1}/f_{X,\mathrm{tg},0}dP_0$. Define D_α^* by $\eta \geq c_\alpha^* \iff f_{X,\mathrm{tg},1}/f_{X,\mathrm{tg},0} \geq D_\alpha^*$, so that equivalently $G^* = \{f_{X,\mathrm{tg},1}/f_{X,\mathrm{tg},0} \geq D_\alpha^*\}$. Then we can rewrite the difference in true positive rate as

$$\begin{aligned}
&P_1(\hat{G}) - P_1(G^*) \\
&= \int \left(\mathbb{1}_{\{\hat{G}\}} - \mathbb{1}_{\{G^*\}}\right) \frac{f_{X,\mathrm{tg},1}}{f_{X,\mathrm{tg},0}} dP_0 \\
&= \int_{(\hat{G}\triangle G^*)\cap \hat{G}} \frac{f_{X,\mathrm{tg},1}}{f_{X,\mathrm{tg},0}} dP_0 - \int_{(\hat{G}\triangle G^*)\cap G^*} \frac{f_{X,\mathrm{tg},1}}{f_{X,\mathrm{tg},0}} dP_0 \\
&= \int_{(\hat{G}\triangle G^*)\cap \hat{G}} \left|\frac{f_{X,\mathrm{tg},1}}{f_{X,\mathrm{tg},0}} - D_\alpha^*\right| + D_\alpha^* dP_0 + \int_{(\hat{G}\triangle G^*)\cap G^*} \left|\frac{f_{X,\mathrm{tg},1}}{f_{X,\mathrm{tg},0}} - D_\alpha^*\right| - D_\alpha^* dP_0 \\
&= \int_{(\hat{G}\triangle G^*)} \left|\frac{f_{X,\mathrm{tg},1}}{f_{X,\mathrm{tg},0}} - D_\alpha^*\right| dP_0 + D_\alpha^* \left(P_0\left(\hat{G} \setminus G^*\right) - P_0\left(G^* \setminus \hat{G}\right)\right) \\
&= \int_{(\hat{G}\triangle G^*)} \left|\frac{f_{X,\mathrm{tg},1}}{f_{X,\mathrm{tg},0}} - D_\alpha^*\right| dP_0 + D_\alpha^* \left(P_0(G^{*c}) - P_0(\hat{G}^c)\right).
\end{aligned}$$

The last equality here is obtained by noticing that G^{*c} is the union of disjoint sets $\hat{G} \setminus G^*$ and $\left(\hat{G} \cup G^*\right)^c$ so that

$$\begin{aligned}
P_0(G^{*c}) - P_0(\hat{G}^c) &= P_0(\hat{G} \setminus G^*) + 1 - P_0(\hat{G}^c) - P_0\left(\hat{G} \cup G^*\right) \\
&= P_0(\hat{G} \setminus G^*) + P_0(\hat{G}) - P_0\left(\hat{G} \cup G^*\right) \\
&= P_0\left(\hat{G} \setminus G^*\right) - P_0\left(G^* \setminus \hat{G}\right).
\end{aligned}$$

In sum,

$$P_1(\hat{G}) - P_1(G^*) = \int_{(\hat{G}\triangle G^*)} \left|\frac{f_{X,\mathrm{tg},1}}{f_{X,\mathrm{tg},0}} - D_\alpha^*\right| dP_0 + D_\alpha^* \left(P_0(G^{*c}) - P_0(\hat{G}^c)\right). \tag{S5.3}$$

Denote the two terms in the RHS of (S5.3) as T_1 and T_2. Note that $\mathbb{E}[P_0(G^{*c}) \mid Y^{\text{tg}} = 0]$ and $\mathbb{E}[P_0(\hat{G}^c) \mid Y^{\text{tg}} = 0]$ are the false positive rates of the true and estimated rule. While we assume that the true rule achieve a false positive rate of α via (P1), so does the empirical false positive rate of the estimated rule utilizing randomized rule if necessary, thus $T_2 = O_p\left(a_n^{(w)} + \sqrt{\log n_c / n_c}\right)$ by Proposition S5.2.

Next we bound term T_1. We start by rewriting the ratio $f_{X,\text{tg},1}/f_{X,\text{tg},0}$ in terms of η. Condition (A3) grants that there is a one-to-one corresponding between the regression function and the ratio of conditional density:

$$f_{X,\text{tg},1}/f_{X,\text{tg},0} = \frac{1-\pi}{\pi}\frac{\eta}{1-\eta},$$

where $\pi = \mathbb{P}\left(Y^{\text{tg}} = 1\right)$. This implies $D_\alpha^* = (1-\pi)c_\alpha^*/(\pi(1-c_\alpha^*))$. Since $0 < \eta \le \sup \eta < 1$, by Lipschitz continuity,

$$\left|\frac{f_{X,\text{tg},1}}{f_{X,\text{tg},0}} - D_\alpha^*\right| \le U_0 \left|\eta - c_\alpha^*\right|$$

with some finite constant $U_0 = (1-\pi)/(\pi(1-\sup\eta)^2) > 0$.

The next step is to bound $\hat{c}_\alpha$ by c_α^*. Following steps similar to that in Proposition S5.2, for arbitrary $\varepsilon > 0$, there exists some constant U_1 such that $\mathbb{P}\left(\cap_{l>0}\bar{\mathcal{E}}_l | \mathcal{D}_\eta\right) \ge 1-\varepsilon$ for all n_c and n_w sufficiently large, where $\bar{\mathcal{E}}_l$ is an event (of $\mathcal{D}_\eta$ and $\mathcal{D}_c$) defined as

$$\bar{\mathcal{E}}_l := \left\{\left|\frac{\sum_{\mathcal{D}_c} \hat{w}_i \mathbb{1}_{\{\hat{\eta}_i > \hat{c}_\alpha - l\}}}{\sum_{\mathcal{D}_c} \hat{w}_i} - P_0\left(\hat{\eta} > \hat{c}_\alpha - l\right)\right| \le U_1\left(a_n^{(w)} + \sqrt{\frac{\log n_c}{n_c}}\right)\right\}.$$

Since $\hat{c}_\alpha$ is infimum, for smaller cutoff value of $\hat{c}_\alpha - l$ with $l > 0$, the empirical false positive rate is always greater than α. This implies

$$P_0\left(\hat{\eta} > \hat{c}_\alpha - l\right) > \alpha - U_1\left(a_n^{(w)} + \sqrt{\frac{\log n_c}{n_c}}\right)$$

on event $\cap_{l>0}\bar{\mathcal{E}}_l$.

By condition (R7), we can define another event (of $\mathcal{D}_\eta$), with $\|\cdot\|_\infty$ taken over $\mathcal{X}_{\text{tg}}$,

$$\mathcal{E} := \left\{\|\hat{\eta} - \eta\|_\infty \le U_2 a_n^{(\eta)}\right\}$$

for some constant $U_2 > 0$ so that $\mathbb{P}\left(\mathcal{E}\right) \ge 1-\varepsilon$ for all sufficiently large n_η. Then on $\mathcal{E} \cap \left(\cap_{l>0}\bar{\mathcal{E}}_l\right)$ whose probability is no smaller than $1-2\varepsilon$, we have

$$\alpha - U_1\left(a_n^{(w)} + \sqrt{\frac{\log n_c}{n_c}}\right) < P_0\left(\hat{\eta} > \hat{c}_\alpha - l\right) \le P_0\left(\eta > \hat{c}_\alpha - l - U_2 a_n^{(\eta)}\right).$$

Further, by condition (P3) we have

$$P_0\left(\eta > c_\alpha^* + t\right) = P_0\left(\eta \ge c_\alpha^*\right) - P_0\left(c_\alpha^* \le \eta \le c_\alpha^* + t\right) \le \alpha - U_1\left(a_n^{(w)} + \sqrt{\frac{\log n_c}{n_c}}\right),$$

with

$$t = \left(\frac{U_1}{C_1}\left(a_n^{(w)} + \sqrt{\frac{\log n_c}{n_c}}\right)\right)^{1/\underline{\gamma}},$$

where C_1 is the constant in the level α detection condition. In combine,

$$P_0\left(\eta > c_\alpha^* + t\right) \le \alpha - U_1\left(a_n^{(w)} + \sqrt{\frac{\log n_c}{n_c}}\right) \le P_0\left(\eta > \hat{c}_\alpha - l - U_2 a_n^{(\eta)}\right)$$

implies

$$c_\alpha^* + t \ge \hat{c}_\alpha - l - U_2 a_n^{(\eta)}$$

for all $l > 0$ given sufficiently large n_w, n_η, and n_c. Since l is arbitrary, we have $\hat{c}_\alpha \le c_\alpha^* + \delta_1$ on $\mathcal{E} \cap (\cap_{l>0} \bar{\mathcal{E}}_l)$, where for simplicity we write

$$\delta_1 = \left(\frac{U_1}{C_1}\left(a_n^{(w)} + \sqrt{\frac{\log n_c}{n_c}}\right)\right)^{1/\underline{\gamma}} + U_2 a_n^{(\eta)}.$$

We now return to T_1 and split $G^{*c} \cap \hat{G} = \{\eta \ge c_\alpha^*, \hat{\eta} < \hat{c}_\alpha\} = A_1 \cup A_2$, where

$$\begin{aligned} A_1 &= \left\{c_\alpha^* \le \eta \le c_\alpha^* + 2\delta_1, \hat{\eta} < \hat{c}_\alpha\right\}, \\ A_2 &= \left\{\eta > c_\alpha^* + 2\delta_1, \hat{\eta} < \hat{c}_\alpha\right\}. \end{aligned}$$

By condition (P2),

$$\int_{A_1} \left|\frac{f_{X,\mathrm{tg},1}}{f_{X,\mathrm{tg},0}} - D_\alpha^*\right| dP_0 \le U_0 \int_{A_1} |\eta - c_\alpha^*|\, dP_0 \le 2U_0\delta_1 P_0\left(A_1\right) \le 2U_0 C_0 \delta_1^{1+\bar{\gamma}},$$

where C_0 is that in the margin assumption. Moreover, note that $A_2 \cap \mathcal{E} \cap (\cap_{l>0} \bar{\mathcal{E}}_l) = \varnothing$, thus

$$\int_{G^{*c} \cap \hat{G}} \left|\frac{f_{X,\mathrm{tg},1}}{f_{X,\mathrm{tg},0}} - D_\alpha^*\right| dP_0 \le 2U_0 C_0 \delta_1^{1+\bar{\gamma}},$$

on $\mathcal{E} \cap (\cap_{l>0} \bar{\mathcal{E}}_l)$. Similar derivation applies to $G^* \cap \hat{G}^c$, so that $T_1 \le 2U_0 C_0 \delta_1^{1+\bar{\gamma}}$ with probability no smaller than $1 - 2\varepsilon$. In sum, we have

$$T_1 = O_p\left(\left(a_n^{(w)} + \sqrt{\frac{\log n_c}{n_c}}\right)^{\frac{1+\bar{\gamma}}{\underline{\gamma}}} + a_n^{(\eta)\,1+\bar{\gamma}}\right),$$

as n_η, n_w, and $n_c \to \infty$. ☐

Proposition S5.4. *Under conditions (A1), (A2), (A3), (A4), (A5), (P1), (R1), (R2), and (R3),*

$$\left|\mathrm{FPR}(\hat{R}) - \alpha\right| = O_p\left(a_n^{(w)} + \sqrt{\log n / n}\right).$$

If further given conditions (P2), (P3), (R4), and (R5), we have

$$\left|\mathrm{TPR}(\hat{R}) - \mathrm{TPR}^*\right| = O_p\left(\left(a_n^{(w)} + \sqrt{\frac{\log n}{n}}\right)^{\min\left\{1, \frac{1+\bar{\gamma}}{\underline{\gamma}}\right\}} + \left(a_n^{(\eta)}\right)^{1+\bar{\gamma}}\right),$$

where $a_n^{(w)} = a_n^{(w,X)} + a_n^{(w,Y)}$, $a_n^{(\eta)} = a_n^{(\tau)} + a_n^{(\Delta)}$, $\hat{R} = \hat{R}(x) = \mathbb{1}_{\{\hat{\eta}(x) > \hat{c}_\alpha\}}$ *is the shift-adjusted rule with* $\hat{\eta}(x) = \hat{w}_Y(x,1)\hat{\tau}(x)$, *and* TPR^* *is the true positive rate for the optimal rule* $\mathbb{1}_{\{\eta(x) \ge c_\alpha^*\}}$.

Proof. Combine Proposition S5.1, Proposition S5.2, and Proposition S5.3. ☐

S6 Uniform Consistency of Nadaraya–Watson Estimator

As the key building stone for estimation, we provide uniform consistency of kernel smoothing. Notation-wise, let $(X_i, Y_i), i = 1, \ldots, n$ be i.i.d. copies of (X, Y). Write $f_{X,Y}$ be the joint density of (X, Y), f_X be the marginal density of X, $f_{X|Y}$ be the conditional density of $X|Y$, and $m(x) = \mathbb{E}[Y_1 \mid X_1 = x]$. The Nadaraya–Watson estimator of m is

$$\hat{m}(x) = \frac{\sum_{i=1}^n Y_i K_h(x - X_i)}{\sum_{i=1}^n K_h(x - X_i)},$$

where $K_h(u) = h^{-1}K(u/h)$.

Lemma S6.1 (Theorem 8, Hansen (2008)). *Under (K1), suppose* $\sup_x f_X(x) < \infty$ *and that the second derivatives of* $f_X(x)$ *and* $f_X(x)m(x)$ *are bounded and uniform continuous. Write* $\delta_n = \inf_{|x|\le c_n} f(x)$ *for some sequence* $c_n > 0$. *If* $n \to \infty, h \to 0$ *in a way such that*

$$\frac{\log n}{nh^d} \to 0, \quad c_n = O\left(n^{1/2q} \log n\right) \text{ for arbitrary } q > 0,$$

and

$$\delta_n^{-1}\left(h^2 + \sqrt{\frac{\log n}{nh^d}}\right) \to 0,$$

then

$$\sup_{|x|\le c_n} |\hat{m}(x) - m(x)| = O_p\left(\delta_n^{-1}\left(h^2 + \sqrt{\frac{\log n}{nh^d}}\right)\right).$$

Remark. Note that when support of f or the domain of interest is compact, c_n is bounded and δ_n can be removed from the rate.

In practice the Nadaraya–Watson estimator is constructed based on the estimated single index $v(\hat{\theta}, X) = \hat{\theta}^T X$, bringing additional bias as characterized by the following result. Similar results can be found in theorem 1 of Chiou and Müller (1998).

Lemma S6.2 (Error-in-variable). *Write* $V = \theta^T X$ *and* $m_\theta(v) = \mathbb{E}[Y \mid \theta^T X = v]$ *given any* $\theta \in \Theta \subset \mathbb{R}^d$. *WLOG, all elements in* θ *are non-zero for all* $\theta \in \Theta$. *Consider the estimator*

$$\hat{m}_\theta(v) = \frac{\sum_{i=1}^n Y_i K_h(v - \theta^T X_i)}{\sum_{i=1}^n K_h(v - \theta^T X_i)}.$$

Under (K1), suppose $\mathcal{X} = \{x : f_X(x) > 0\}$ *and* Θ *are compact, and that the first two derivatives of* $f_X(x)$ *and* $f_X(x)\,\mathbb{E}[Y \mid X = x]$ *are uniform continuous. If* $n \to \infty, h \to 0$ *in a way such that*

$$\frac{\log n}{nh} \to 0,$$

then

$$\sup_{\theta\in\Theta, x\in\mathcal{X}} \left|\hat{m}_\theta(\theta^T x) - m_\theta(\theta^T x)\right| = O_p\left(h^2 + \sqrt{\frac{\log n}{nh}}\right). \quad \text{(S6.1)}$$

Further,

$$\sup_{x\in\mathcal{X}}\left|\hat{m}_\theta(\theta^T x) - m_{\theta_0}({\theta_0}^T x)\right| = O_p\left(h^{-1}\|\theta-\theta_0\| + h^2 + \sqrt{\frac{\log n}{nh}}\right) \tag{S6.2}$$

for any sequence of θ *and bandwidth* h *such that* $h^{-1}\|\theta-\theta_0\| = o_p(1)$*.*

Proof. By change of variables, we have that the density function of $V=\theta^T X$ is $f_V(v;\theta) = \int f_X(u_1/\theta_1,\ldots,(v-u_{d-1})/\theta_d)du_1\ldots du_{d-1}$. Therefore the boundedness and continuity for second derivatives of f_V and $f_V m_\theta$ inherit that of f_X and $f_X m$ under compact $\mathcal{X}$. Then follows the same proof as in theorem 8 of Hansen (2008), write $g(v;\theta) = f_V(v;\theta)m_\theta(v)$. It suffices to, for

$$\hat{f_V}(\theta^T x;\theta) = \frac{1}{nh}\sum_{i=1}^n K_h\left(\theta^T(x-X_i)\right),$$

bound

$$\sup_{\theta,x}\left|\hat{f_V}(\theta^T x;\theta) - \mathbb{E}[\hat{f_V}(\theta^T x;\theta)]\right|$$

and

$$\sup_{\theta,x}\left|\mathbb{E}[\hat{f_V}(\theta^T x;\theta)] - f_V(\theta^T x;\theta)\right|;$$

so as for $\hat{g}(\theta^T x;\theta) = \frac{1}{nh}\sum_{i=1}^n Y_i K_h(\theta^T x - \theta^T X_i)$.

For the first part, note that $n\to\infty, h\to 0$, and $\log n/(nh)\to 0$ also implies $|\log h|/(nh)\to 0$. Therefore it suffices to consider the VC-class of functions

$$\mathcal{K}_h = \left\{x\mapsto K\left(\frac{\theta^T(t-x)}{h}\right) \text{ for } x\in\mathcal{X} : t\in\mathcal{X},\theta\in\Theta\right\}$$

index by $t\in\mathcal{X},\theta\in\Theta$. By change of variable, for all $f\in\mathcal{K}_h$,

$$\begin{aligned}
&\operatorname{var} f(X) \le \mathbb{E}[f(X)^2] \\
&= \int K\left(\frac{\theta^T t - v}{h}\right)^2 \int f_X\left(\frac{u_1}{\theta_1},\ldots,\frac{u_{d-1}-u_{d-2}}{\theta_{d-1}},\frac{v-u_{d-1}}{\theta_d}\right)\frac{du_1\cdots du_{d-1}}{\theta_1\cdots\theta_d}dv \\
&\lesssim h\|K\|_\infty^2\|f_X\|_\infty,
\end{aligned}$$

where u_j,θ_j are the j-th element of d-dimensional vectors u,θ for $j=1,\ldots,d$. and $\|f\|_\infty \le \|K\|_\infty$. Henec, the Talagrand's inequality (e.g., Giné and Guillou, 2002, corollary 2.2) implies

$$\sup_{\theta,x}\left|\hat{f_V}(\theta^T x;\theta) - \mathbb{E}[\hat{f_V}(\theta^T x;\theta)]\right| = O_p\left(\sqrt{\frac{\log n}{nh}}\right).$$

As for the bias part, by differentiability, we have

$$\begin{aligned}
&\mathbb{E}[K_h\left(\theta^T(t-X)\right)] - f_V(\theta^T t;\theta) \\
&= \int K(v)\left(f_V(\theta^T t - vh;\theta) - f_V(\theta^T t;\theta)\right)dv \\
&= \int K(v)\left(-vhf_V'(\theta^T t - vh;\theta) + \frac{(vh)^2}{2}f_V''(\tilde{v};\theta)\right)dv
\end{aligned}$$

with some $\tilde{v}$ between $\theta^T t - vh$ and $\theta^T t$. Note that

$$f'_V(v;\theta) = \theta_d^{-1} \int \partial_d f_X \left(\frac{u_1}{\theta_1}, \ldots, \frac{u_{d-1} - u_{d-2}}{\theta_{d-1}}, \frac{v - u_{d-1}}{\theta_d} \right) \frac{du_1 \cdots du_{d-1}}{\theta_1 \cdots \theta_d},$$
$$f''_V(v;\theta) = \theta_d^{-2} \int (\partial_d)^2 f_X \left(\frac{u_1}{\theta_1}, \ldots, \frac{u_{d-1} - u_{d-2}}{\theta_{d-1}}, \frac{v - u_{d-1}}{\theta_d} \right) \frac{du_1 \cdots du_{d-1}}{\theta_1 \cdots \theta_d},$$

where ∂_d means taking partial derivative w.r.t. the d-th argument. Therefore, $\sup_{v,\theta} |f'_V(v;\theta)|$ and $\sup_{v,\theta} |f''_V(v;\theta)|$ are bounded by the uniform continuity of f'_X and f''_X. Thus, under (K1), $\int vK(v)dv = 0$ and $\int v^2 K(v)dv < \infty$, thus

$$\sup_{\theta,t} \left| \mathbb{E}[K_h\left(\theta^T(t - X)\right)] - f_V(\theta^T t;\theta) \right| = O_p\left(h^2\right).$$

Repeats the same steps for $\hat{g}$ gives (S6.1).

As for (S6.2), it suffices to consider the bias term as

$$\mathbb{E}[\hat{g}(\theta^T x) - g(\theta_0^T x)] = \mathbb{E}[\hat{g}(\theta^T x) - \hat{g}(\theta_0^T x)] + \mathbb{E}[\hat{g}(\theta_0^T x) - g(\theta_0^T x)],$$

where we omit the additional θ so $\hat{g}(\theta^T x)$ and $g(\theta^T x)$ are understood as $\hat{g}(\theta^T x;\theta)$ and $g(\theta^T x;\theta)$ respectively, so as for θ_0.

Obviously, the last term is the usual bias for kernel smoothing, so $\mathbb{E}[\hat{g}(\theta_0^T x) - g(\theta_0^T x)] = O_p\left(h^2\right)$. Further

$$\begin{aligned}
&\mathbb{E}[\hat{g}(\theta^T x) - \hat{g}(\theta_0^T x)] \\
&= \mathbb{E}[Y_1\left(K_h(\theta^T(x - X_1)) - K_h(\theta_0^T(x - X_1))\right)] \\
&\lesssim_p h^{-1}\,\mathbb{E}[Y_1\left(K'\left(\frac{\theta_0^T(x - X_1)}{h}\right) h^{-1}\|\theta - \theta_0\| + O_p\left(h^{-2}\|\theta - \theta_0\|^2\right)\right)] \\
&\lesssim_p h^{-1}\int m_{\theta_0}(v) K'\left(\frac{\theta_0^T x - v}{h}\right)\left(h^{-1}\|\theta - \theta_0\| + O_p\left(h^{-2}\|\theta - \theta_0\|^2\right)\right) f_V(v)dv \\
&\asymp_p h^{-1}\|\theta - \theta_0\| \int m_{\theta_0}(\theta_0^T x - uh)K'(u) f_V(\theta_0^T x - uh)du + O_p\left(h^{-2}\|\theta - \theta_0\|^2\right) \\
&= O_p\left(h^{-1}\|\theta - \theta_0\|\right).
\end{aligned}$$

□

S7 Analysis of the Plug-in Estimators

S7.1 Single Index Model

We now present some propositions for consistency of the single index model estimation. Let $X_i^{\mathrm{sr}}, Y_i^{\mathrm{sr}}$, $i = 1, \ldots, n$ be i.i.d. random sample drawn from source population. (I.e., from model (6) with $S_i = 1$.)

Proposition S7.1 (theorem 5.2, Ichimura (1993))**.** *Suppose*

- *(S1) θ_0 is in the interior of Θ, Θ is compact. φ is differentiable and not a constant function on the support of ${\theta_0}^T X^{\mathrm{sr}}$.*
- *(S2) The set $A_\delta := \left\{x : f_{V,\mathrm{sr}}(\theta^T x;\theta) \geq \delta,\ \text{for all } \theta \in \Theta\right\}$ for some constant δ is compact. $\inf_x f_{V,\mathrm{sr}}(\theta^T x;\theta) > 0$*

(S3) On $\{\theta^T x : \theta \in \Theta, x \in A_\delta\}$ the $f_{V,\mathrm{sr}}(v;\theta)$ and $v \mapsto \mathbb{E}[Y^{\mathrm{sr}} \mid \theta^T X^{\mathrm{sr}} = v]$ are three times continuously differentiable with Lipschitz continuous (in v) third derivatives uniformly over $\theta \in \Theta$.

suppose $n \to \infty$ and the bandwidth $h_\varphi \to 0$ in the sense that $|\log h_\varphi| / (nh_\varphi^4) \to 0$ and $nh_\varphi^8 \to 0$, then under (K1),

$$\|\hat{\theta} - \theta_0\|_\infty = O_p\left(\frac{1}{\sqrt{n}}\right).$$

Proof. See, e.g., theorem 5.2 of Ichimura (1993); theorem 4 of Klein and Spady (1993); theorem 8.1 of Li and Racine (2007); theorem 4.1 of Delecroix et al. (2006). □

Corollary S7.1. *Under (A1), (A2), (A3), conditions (S1)–(S3) are satisfied. Suppose $n \to \infty$ and the bandwidth $h_\varphi \to 0$ in the sense that $|\log h_\varphi| / (nh_\varphi^4) \to 0$ and $nh_\varphi^8 \to 0$, then under (K1),*

$$\sup_{x \in \mathcal{X}} \left|\hat{\varphi}\left(\hat{\theta}^T x\right) - \varphi\left({\theta_0}^T x\right)\right| = O_p\left(\frac{1}{\sqrt{n} h_\varphi} + h_\varphi^2 + \sqrt{\frac{\log n}{nh_\varphi}}\right),$$

and for $\hat{\varphi}(v;\theta)$ based on $\theta^T X_i^{\mathrm{sr}}$,

$$\sup_{\theta \in \Theta} \sup_{v \in \mathcal{V}_\theta} \left|\hat{\varphi}(v;\theta) - \mathbb{E}[Y^{\mathrm{sr}} \mid \theta^T X^{\mathrm{sr}} = v]\right| = O_p\left(h_\varphi^2 + \sqrt{\frac{\log n}{nh_\varphi}}\right).$$

Proof. Use Lemma S6.2. For the second rate, it suffices to notice $\mathcal{V}_\theta$ is irrelevant to the rates in (S6.1). □

An implication of Corollary S7.1 is a bound for $\sup_{v \in \mathcal{V}_{\hat{\theta}}} |\hat{\varphi}(v) - \varphi(v)|$. For $v \in \mathcal{V}_{\hat{\theta}}$ and some $x \in \mathcal{X}$,

$$\begin{aligned}
&|\hat{\varphi}(v) - \varphi(v)| \\
&= \left|\hat{\varphi}(\hat{\theta}^T x) - \varphi(\hat{\theta}^T x)\right| \\
&\leq \left|\hat{\varphi}(\hat{\theta}^T x) - \varphi({\theta_0}^T x)\right| + \left|\varphi({\theta_0}^T x) - \varphi(\hat{\theta}^T x)\right| \\
&\leq \left|\hat{\varphi}(\hat{\theta}^T x) - \varphi({\theta_0}^T x)\right| + \|\varphi'\|_\infty \left|(\hat{\theta} - \theta_0)^T x\right| \\
&= O_p\left(\frac{1}{\sqrt{n} h_\varphi} + h_\varphi^2 + \sqrt{\frac{\log n}{nh_\varphi}}\right).
\end{aligned}$$

S7.2 Estimated Baseline Shift

Suppose $\mathbb{P}(Y^{\mathrm{tg}} = 1)$ is known, let $\tilde{\Delta}_0$ be the solution to

$$\frac{1}{n_\Delta} \sum_{i=1}^{n_\Delta} \varphi\left(\tilde{\Delta}_0 + {\theta_0}^T X_i^{\mathrm{sr}}\right) w_V\left({\theta_0}^T X_i^{\mathrm{sr}}\right) - \mathbb{P}\left(Y^{\mathrm{tg}} = 1\right) = 0, \tag{S7.1}$$

where φ and θ_0 are those in model (6). Note that $w_V = 0$ for $X_i^{\mathrm{sr}} \in \mathcal{X} - \mathcal{X}_{\mathrm{tg}}$. Hence for simplicity we assume $X_i^{\mathrm{sr}} \in \mathcal{D}_{\mathrm{sr}} \cap \mathcal{X}_{\mathrm{tg}}$, and let n_Δ denotes its size. Therefore condition (A5) ensures (S7.1) is well-defined.

Proposition S7.2. *Under (A5), if φ is almost surely (w.r.t. $f_{X,\mathrm{sr}}$) strictly monotone and differentiable with bounded derivative. Suppose $\|w_V\|_\infty$ is finite, then $\sqrt{n_\Delta}\left(\tilde{\Delta}_0 - \Delta_0\right)$ converges in distribution to some mean zero normal random variable as $n \to \infty$.*

Proof. It suffices to verify the conditions (A) – (F) in theorem 13.4 of Kosorok (2008). Following notations there, let ψ_Δ be some function indexed by Δ:

$$\psi_\Delta : v \mapsto \varphi(\Delta + v) w_V(v; \theta_0) - \mathbb{P}\left(Y^{\mathrm{tg}} = 1\right) \text{ for } x \in \mathcal{X}.$$

The distribution of X^{sr} and empirical process $X_1^{\mathrm{sr}}, \ldots, X_{n_\Delta}^{\mathrm{sr}}$ induce

$$\Psi(\Delta) = P\psi_\Delta = \int \varphi(\Delta + v) w_V(v; \theta_0) f_{V,\mathrm{sr}}(v; \theta_0) dv - \mathbb{P}\left(Y^{\mathrm{tg}} = 1\right),$$

$$\Psi_{n_\Delta}(\Delta) = P_n \psi_\Delta = \frac{1}{n_\Delta} \sum_{i=1}^{n_\Delta} \varphi\left(\Delta + {\theta_0}^T X_i^{\mathrm{sr}}\right) w_V\left({\theta_0}^T X_i^{\mathrm{sr}}\right) - \mathbb{P}\left(Y^{\mathrm{tg}} = 1\right).$$

The monotonicity of φ implies Ψ is also (strictly) monotone, so that $\Psi(\Delta) = 0$ only if $\Delta = \Delta_0$. Thus $\Psi(\Delta_n) \to 0$ only if the sequence Δ_n satisfies $|\Delta_n - \Delta_0| \to 0$ as $n \to \infty$, granting condition (A).

Also $\left\{\psi_\Delta : |\Delta| \leq \bar{\Delta}_0 *\right\}$ is a Donsker class for some finite $\bar{\Delta}_0 * > 0$, since

$$|\psi_{\Delta_1}(v) - \psi_{\Delta_2}(v)| \leq \varphi'(u^* + v) w_V(v; \theta_0) |\Delta_1 - \Delta_2|$$

for some u^* between Δ_1 and Δ_2. Now that φ' is bounded, thus by theorem 2.3 and the proceeding discussion in Kosorok (2008), it is a Donsker class and hence Glivenko-Cantelli class, satisfying conditions (B) and (C).

Next, condition (D) is satisfied noting that

$$P(\psi_\Delta - \psi_{\Delta_0})^2 = \mathbb{E}\left(w_V({\theta_0}^T X^{\mathrm{sr}}) \left(\varphi\left(\Delta + {\theta_0}^T X^{\mathrm{sr}}\right) - \varphi\left(\Delta_0 + {\theta_0}^T X^{\mathrm{sr}}\right)\right)^2\right) \to 0$$

for any Δ such that $|\Delta - \Delta_0| \to 0$, given Lipschitz continuity of φ and $\|w_V\|_\infty < \infty$.

We only need to validate the first part of condition (E) since no bootstrap is involved, while essentially requires $\Psi_n(\tilde{\Delta}_0)$ is sufficiently small. Since $\Psi(\Delta_0) = 0$ and by classic C.L.T. $\Psi_{n_\Delta}(\Delta_0) = O_p\left(1/\sqrt{n_\Delta}\right)$. Hence by the strict monotonicity of φ we can always find $\tilde{\Delta}_0$ so that $\Psi_{n_\Delta}(\tilde{\Delta}_0) = o_p\left(1/\sqrt{n_\Delta}\right)$.

In the end, Ψ inherits the differentiability of φ. Thus by theorem 13.4 of Kosorok (2008) we have the desired result. □

Corollary S7.2. *Under (A3) and the conditions of Proposition S7.2,*

$$\left|\hat{\Delta}_0 - \Delta_0\right| \lesssim_p \frac{1}{\sqrt{n_\Delta}} + \sup_x \|\hat{w}_V(\hat{\theta}^T x) - w_V({\theta_0}^T x)\|_\infty + \sup_{v \in \mathcal{V}_{\hat{\theta}}} |\hat{\varphi}(v) - \varphi(v)| + \left|\hat{\theta} - \theta_0\right|.$$

Proof. Write

$$\hat{\Psi}_{n_\Delta}(\Delta) = \frac{1}{n_\Delta} \sum_{i=1}^{n_\Delta} \hat{\varphi}\left(\Delta + \hat{\theta}^T X_i^{\mathrm{sr}}\right) \hat{w}_V\left(\hat{\theta}^T X_i^{\mathrm{sr}}\right) - \mathbb{P}\left(Y^{\mathrm{tg}} = 1\right).$$

Further,

$$\begin{aligned} &\hat{\varphi}\left(\Delta + \hat{\theta}^T x\right) - \varphi\left(\Delta + {\theta_0}^T x\right) \\ &= \hat{\varphi}\left(\Delta + \hat{\theta}^T x\right) - \varphi\left(\Delta + \hat{\theta}^T x\right) + \varphi\left(\Delta + \hat{\theta}^T x\right) - \varphi\left(\Delta + {\theta_0}^T x\right), \end{aligned}$$

together with (A3) so that φ is strictly monotone and differentiable with bounded derivative, thus

$$\sup_{\Delta}\left|\hat{\Psi}_{n_\Delta}(\Delta)-\Psi_{n_\Delta}(\Delta)\right| \lesssim \sup_x \|\hat{w}_V(\hat{\theta}^T x)-w_V({\theta_0}^T x)\|_\infty + \sup_{v\in\mathcal{V}_{\hat{\theta}}} |\hat{\varphi}(v)-\varphi(v)| + \left|\hat{\theta}-\theta_0\right|.$$

Further note that Ψ_{n_Δ} is invertable with differentiable inverse. Thus $\hat{\Delta}_0-\tilde{\Delta}_0 = O_p\left(\Psi_{n_\Delta}(\hat{\Delta}_0)-\Psi_{n_\Delta}(\tilde{\Delta}_0)\right)$. The result follows from Proposition S7.2 by noting $\Psi_{n_\Delta}(\tilde{\Delta}_0)$ and $\hat{\Psi}_{n_\Delta}(\hat{\Delta}_0)$ are both arbitrarily small for sufficiently large n_Δ. □

S7.3 Proof of Theorem 3.1

Proposition S7.3. *Under conditions (A1), (A2), (A3), (A4), (K1), and (B1), we have* $\|\hat{\theta}-\theta_0\|_\infty = O_p(1/\sqrt{n})$, *and that (R2) and (R4) are satisfied with*

$$a_n^{(w,X)} = h_\psi^2 + \sqrt{\frac{\log n}{nh_\psi}}, \quad a_n^{(\tau)} = \frac{1}{\sqrt{n}h_\varphi} + h_\varphi^2.$$

Moreover,

$$\sup_{x\in\mathcal{X}}\left|\hat{w}_V(\hat{\theta}^T x)-w_V({\theta_0}^T x)\right| = O_p\left(\frac{1}{\sqrt{n}h_\psi}+h_\psi^2+\sqrt{\frac{\log n}{nh_\psi}}\right).$$

Proof. Use Proposition S7.1 and Corollary S7.1 for $\hat{\theta}$ and $\hat{\tau}$. Indeed, it suffices to note that

$$(\sqrt{n}h_\varphi)\sqrt{\log n/(nh_\varphi)} = \sqrt{h_\varphi \log n} \to 0$$

given $nh_\varphi^8 \to 0$ in (B1). For the uniform rate of $\hat{w}_V$, note that the marginal density of V (pooling $S=0,1$) is a convex combination of $f_{V,\mathrm{sr}}$ and $f_{V,\mathrm{tg}}$ with non-zero coefficients, thus inherits the smoothness conditions (A2). Under (A1), (A2), and (A4), conditions in Lemma S6.2 are satisfied by change of variables, leading to the stated rates. □

Combining Proposition S7.3 and Corollary S7.2 leads to the rate of $\hat{\Delta}_0$ in Proposition 3.1. Note that $\sqrt{n}h_\psi = o(\sqrt{n})$.

Proof of Theorem 3.1. To verify (R5), note that by (9) and that φ is bounded and continuously differentiable, given any $x\in\mathcal{X}_{\mathrm{tg}}$, $\left|\hat{w}_Y\left(\hat{\theta}^T x, y; \hat{\theta}\right) - w_Y\left({\theta_0}^T x, y; \theta_0\right)\right| \lesssim \left|\hat{\varphi}\left(\hat{\Delta}_0+\hat{\theta}^T x\right)-\varphi\left(\hat{\Delta}_0+\hat{\theta}^T x\right)\right| + \left|\varphi\left(\hat{\Delta}_0+\hat{\theta}^T x\right)-\varphi\left(\Delta_0+{\theta_0}^T x\right)\right|$, where the first term is bounded by $\sup_{v\in\mathcal{V}_{\hat{\theta}}}|\hat{\varphi}(v)-\varphi(v)| = O_p(b_n)$ by Corollary S7.1, while the last term is asymptotically bounded by $\left|\hat{\Delta}_0-\Delta_0\right|+\left|\hat{\theta}-\theta_0\right|$. Therefore (R5) is satisfied by taking $a_n^{(\Delta)} = b_n$.

In the end, we validate (R3) with $a_n^{(w,Y)} = b_n$ and $\Theta_n = \left\{\theta\in\Theta : \|\theta-\theta_0\|_\infty \le \|\hat{\theta}_n-\theta_0\|_\infty\right\}$ by showing $\sup_{v\in\mathcal{V},\theta\in\Theta_n}\left|\hat{\varphi}\left(\hat{\Delta}_0+v\right)-\mathbb{E}[Y^{\mathrm{tg}}\mid \theta^T X^{\mathrm{tg}}=v]\right| = O_p(b_n)$. By change of variable,

$$\begin{aligned}&\mathbb{E}[Y^{\mathrm{tg}}\mid\theta^T X^{\mathrm{tg}}=v] f_{V,\mathrm{tg}}(v;\theta)\\ &= \int \varphi\left(\Delta_0+\sum_{j=2}^d\left(\frac{\theta_{0,j}}{\theta_j}-\frac{\theta_{0,j-1}}{\theta_{j-1}}\right)u_j + \frac{\theta_{0,d}}{\theta_d}v\right) f_{X,\mathrm{tg}}\left(\frac{u_1}{\theta_1},\cdots,\frac{v-u_{d-1}}{\theta_d}\right)\frac{du_1\cdots du_{d-1}}{\theta_1\cdots\theta_d},\end{aligned}$$

where θ_j and $\theta_{0,j}$ are the j-th element of θ and θ_0, while the marginal density of $\theta^T X^{\text{tg}}$ is $f_{V,\text{tg}}(v;\theta) = \int f_{X,\text{tg}}\left(\frac{u_1}{\theta_1}, \cdots, \frac{v-u_{d-1}}{\theta_d}\right) du_1 \cdots du_{d-1} / (\theta_1 \cdots \theta_d)$, with integration taken over $(u_1, \ldots, u_{d-1})$ with $u_1/\theta_1 \in \mathcal{X}_{\text{tg}}, (u_j - u_{j-1})/\theta_j \in \mathcal{X}_{\text{tg}}, j = 2, \ldots, d-1$. Therefore it suffices to consider

$$\left|\hat{\varphi}\left(\hat{\Delta}_0 + v\right) - \varphi\left(\Delta_0 + \sum_{j=2}^{d}\left(\frac{\theta_{0,j}}{\theta_j} - \frac{\theta_{0,j-1}}{\theta_{j-1}}\right)u_j + \frac{\theta_{0,d}}{\theta_d}v\right)\right|$$
$$\leq \sup_v |\hat{\varphi}(v) - \varphi(v)| + \|\varphi'\|_\infty \left|\hat{\Delta}_0 - \Delta_0 - \sum_{j=2}^{d}\left(\frac{\theta_{0,j}}{\theta_j} - \frac{\theta_{0,j-1}}{\theta_{j-1}}\right)u_j + \left(1 - \frac{\theta_{0,d}}{\theta_d}\right)v\right|,$$

which is $O_p(b_n)$ uniformly over $\theta \in \Theta_n$. □

Remark (w_X-weighting)**.** The theoretical treatment can be greatly simplified by replacing w_V with w_X, as no error-in-variable type bias remains. Following a similar derivation, the final rates for TPR and FPR are

$$h_\psi^2 + \frac{1}{\sqrt{n}} + \sqrt{\log n/(nh_\psi^d)} + h_\varphi^2 + \frac{1}{\sqrt{nh_\varphi}}$$

but replacing $1/\sqrt{n}h$ terms by $1/\sqrt{n}$, and $\sqrt{\log n/(nh)}$ terms by $\sqrt{\log n/(nh^d)}$ in b_n of Proposition 3.1. However, it suffers from the dimensionality of X, while w_V is dimensionality invariant. In practice, while estimating θ_0 and φ for model (6) could still remain challenging for high-dimensional cases, it is believed easier compared to otherwise a high-dimensional propensity model thanks to the additional structure of the linear index.

S8 Implementation Notes

The (semi-)non-parametric estimates for the response model and the propensity model rely on the bandwidth h_y and h_ψ. The former h_y can be chosen following traditional single index model methods; we refer to Delecroix et al. (2000) and references therein. We use the implementation of Klein and Spady (1993) in the R package `np`.

Kullback–Leibler divergence based objective function (Sugiyama et al., 2007) is employed to tune the bandwidth h_ψ in the propensity model. We choose the bandwidth that minimizes $D_{\text{KL}}(f_{\text{tg}} \parallel \hat{w}_X f_{\text{sr}}) \propto -\sum_{i=1}^{n_{\text{tg}}} \hat{w}_{x,-i}(X_i^{\text{tg}}, h_\psi) + o_p(1)$, where $\hat{w}_{x,-i}(X_i^{\text{tg}}, h_\psi)$ is the leave-one-out version of $\hat{w}_X$ leaving out X_i^{tg} that depends on h_ψ through $\hat{\psi}$. We replace the leave-one-out scheme with 10-fold cross-validation to save computation time. Similarly the L^2 error between w_X and $\hat{w}_X$ under f_{sr} can also be used (Kanamori et al., 2009).

We use `emplik`[1] to compute empirical likelihood.

S9 Simulation Details

Combining different φ functions and covariate shifts as listed below constitutes nine simulation setups, each of which was repeated 100 times with $n_{\text{sr}} = 750$ and $n_{\text{tg}} = 100$. The performance was evaluated on another target sample of size 10,000. We set the single index $V = X_1 + X_2$, and considered the following three functions for φ (also pictured in Figure S9.1).

[1] https://artowen.su.domains/empirical/

1. Logistic: $\varphi(v) = \text{logit}^{-1}(v)$;
2. bump:
$$\varphi(v) = \begin{cases} \text{logit}^{-1}(v-1) & , \text{ if } v \le v_l, \\ 0.1 + 0.85\sqrt{1-(v/4-3/4)^2} & , \text{ if } v_l < v \le v_r, \\ \text{logit}^{-1}(v+\text{logit}(0.95)-3) & , \text{ if } v > v_r, \end{cases} \tag{S9.1}$$
for $v_r = 3$ and $v_l \approx -0.999$, which essentially glues a circle to the sigmoid function between -1 and 3.
3. contaminated: $\varphi(v) = \text{logit}^{-1}(v) + 4\phi(2(v+1))/3$ where ϕ is the density of standard normal distribution.

Covariates from the source population were drawn from a normal distribution: $X_i^{\text{sr}} = (X_{i1}^{\text{sr}}, X_{i2}^{\text{sr}})$ with $X_{i1}^{\text{sr}} \perp\!\!\!\perp X_{i2}^{\text{sr}}$, each following a normal distribution with zero mean and variance 4, independently for $i = 1, \ldots, n_{\text{sr}}$. For the target population, one could take one of the following (also pictured in Figure S9.2).

1. squeeze: $\left(X_{i1}^{\text{tg}}, X_{i2}^{\text{tg}}\right)$ follows a bivariate normal distribution with mean zero, $\text{var}\, X_{i1}^{\text{tg}} = \text{var}\, X_{i2}^{\text{tg}} = 3/4$, and $\text{cov}\left(X_{i1}^{\text{tg}}, X_{i2}^{\text{tg}}\right) = -1/4$;
2. mixture: $\left(X_{i1}^{\text{tg}}, X_{i2}^{\text{tg}}\right)$ is drawn from a Gaussian mixture, where with probability 0.2 it is identically distributed as X^{sr}, otherwise follows a bivariate normal distribution with $\mathbb{E}X_{i1}^{\text{tg}} = \mathbb{E}X_{i2}^{\text{tg}} = 1$, $\text{var}\, X_{i1}^{\text{tg}} = \text{var}\, X_{i2}^{\text{tg}} = 1/4$, and $X_{i1}^{\text{tg}} \perp\!\!\!\perp X_{i2}^{\text{tg}}$;
3. ripple: the density for $\left(X_{i1}^{\text{tg}}, X_{i2}^{\text{tg}}\right)$ is $f_{\text{tg}}(x_1, x_2) = 0.8\frac{\phi(2(r-2))}{\pi r} + 0.2 \times 16\phi(4x_1)\phi(4x_2)$, where $r = \sqrt{x_1^2 + x_2^2}$, ϕ is the density of univariate standard normal distribution.

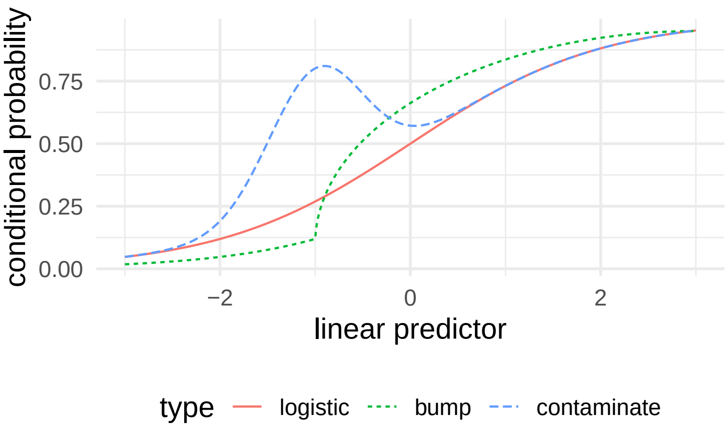

Figure S9.1: φ for simulation.

Figure S9.3 shows the performance while controlling the FPR instead of controlling the TPR as shown in Figure 1 of the main text.

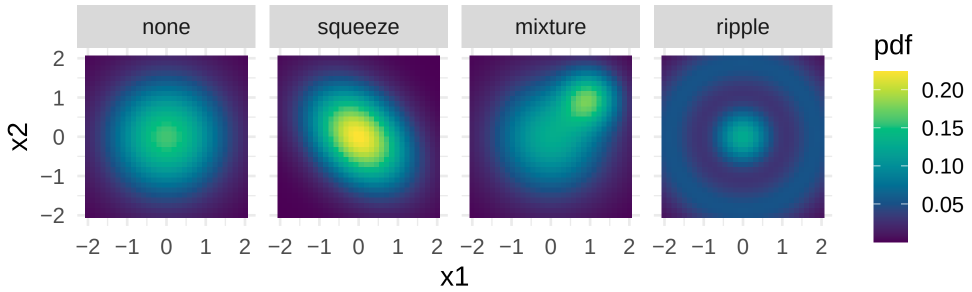

Figure S9.2: Illustrating X-shift: the covariate distributions of the source (left-most "none" panel) and the three target populations.

S9.1 Logistic regression vs single index model

To further clarify our use of the single index model in (6), we conducted an additional simulation comparing the proposed procedure with an otherwise identical implementation in which the single index working model was replaced by standard logistic regression. The two methods share the same framework, weighting scheme, and decision rule construction; the only difference is the model used for the conditional risk $\mathbb{P}(Y = 1|X)$.

Figure S9.4 shows that, when the true risk model is non-logistic, the single index version yields smaller errors in both control of TPR and optimization of FPR. This supports our use of the single index model as a semiparametric compromise: it retains a low-dimensional summary through ${\theta_0}^T X$ while avoiding a restrictive link specification, and is therefore more robust to risk model misspecification. When the logistic specification is approximately correct, the two approaches perform similarly.

S9.2 Computation time

To further examine the computational efficiency of the proposed method, we extended one of the simulation scenarios by increasing the number of covariates and varying the source sample size. Under each configuration, we ran the proposed method and recorded the computation time for the main components of the procedure: (i) fitting the single index model; (ii) fitting the propensity model and computing the weights; and (iii) calculating the cutoff threshold c. Figure S9.5 summarizes these results and indicates that the single index estimation step is the primary computational bottleneck, while the subsequent steps are relatively insensitive to the covariate dimension. This is because it requires repeated nonparametric kernel smoothing to update the link function whenever the index coefficients θ_0 are updated. By contrast, the subsequent steps operate only on the estimated one-dimensional single index $V \in \mathbb{R}$, and are thus insensitive to the number of original covariates.

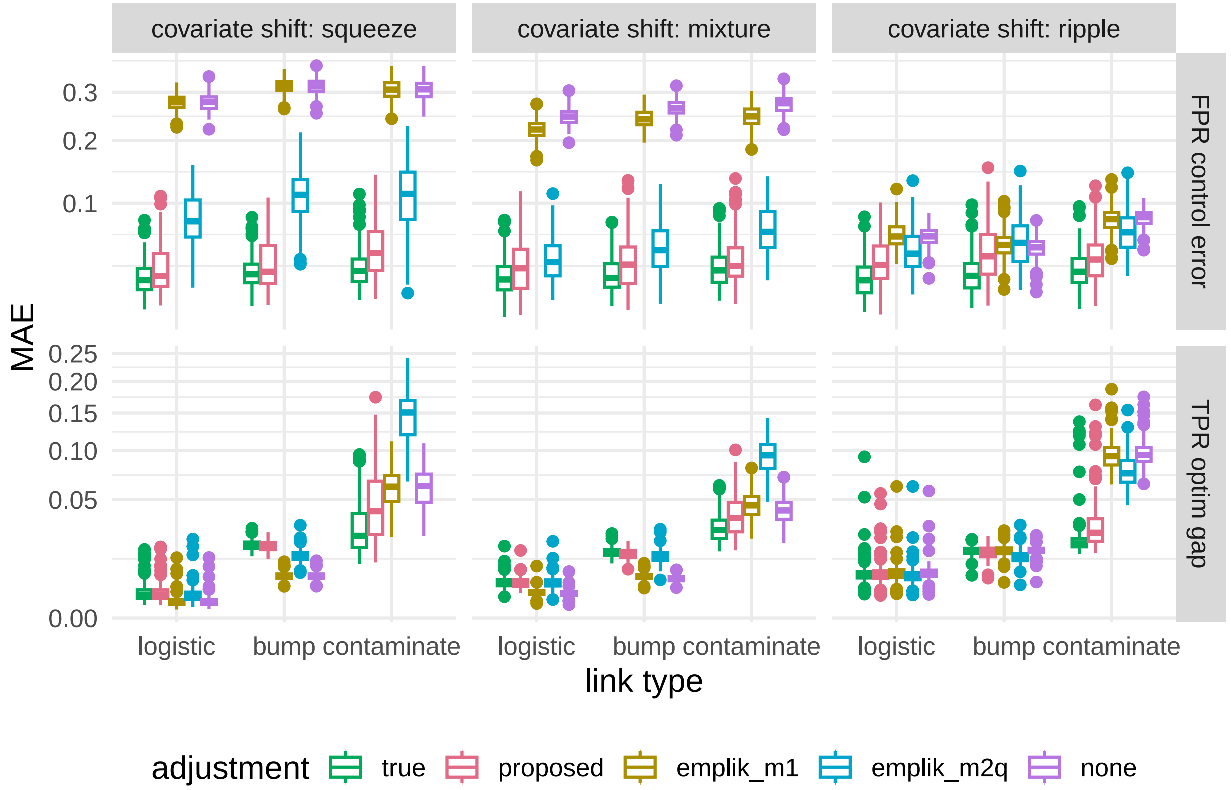

Figure S9.3: In analogy to Figure 1 of the main text, shift-adjustment performance in terms of controlling the FPR while minimizing the TPR, under weighting by the true ratio, the proposed SACSIM, empirical likelihood (emplik_m1 and emplik_m2q), and no adjustment (none). Each dot represents one repeat; figures show 200 repeated experiments.

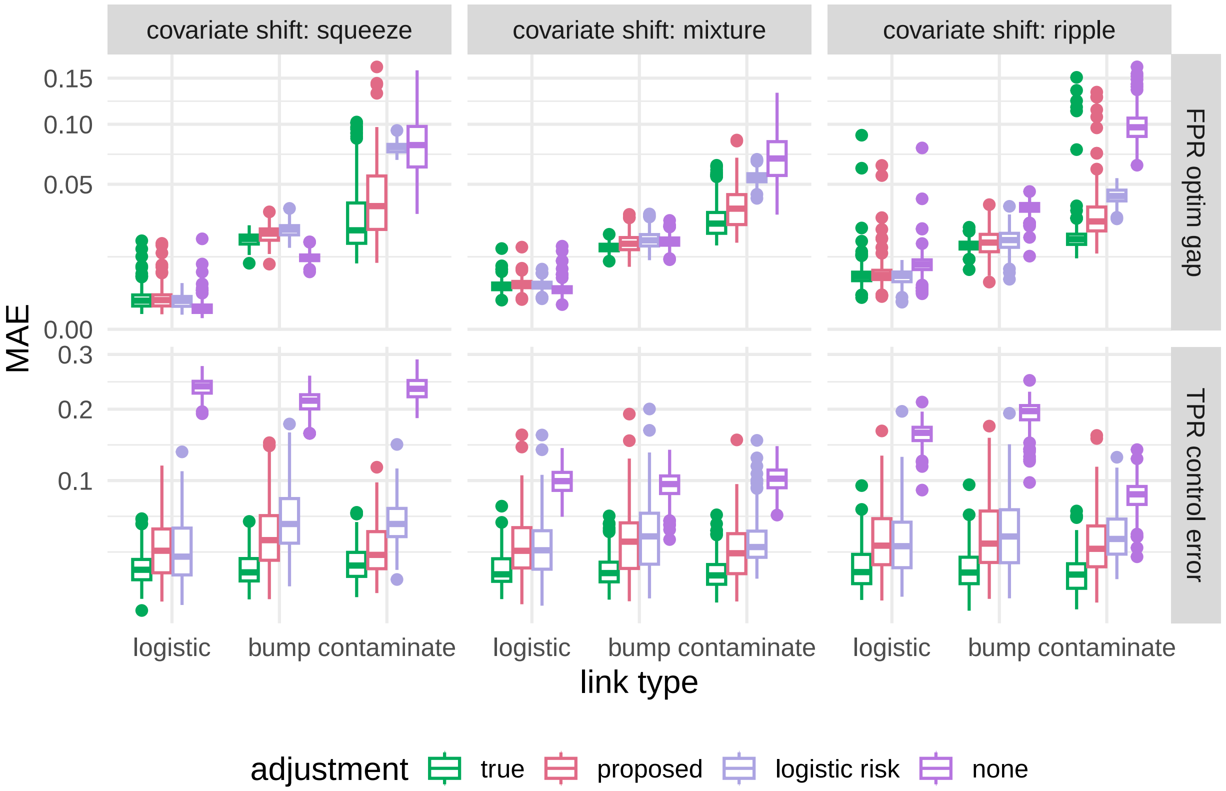

Figure S9.4: In analogy to Figure 1 of the main text, comparison of the proposed method using the single index model in (6) (proposed) and the same procedure with the single index component replaced by logistic regression (logistic risk). Each dot represents one repeat; figures show 200 repeated experiments.

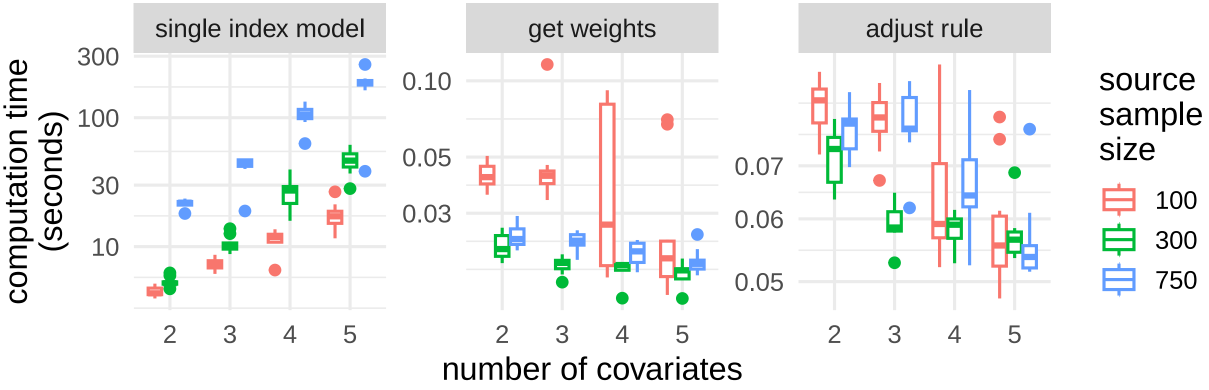

Figure S9.5: Computation time in a simulation study for fitting the single index model (leftmost), fitting the propensity model and computing the weights (middle), and calculating the cutoff threshold c, under varying sample sizes and covariate dimensions. Each dot corresponds to one of 10 repeated experiments.

S9.3 Impact of Assumption Violation

Figure S9.6 examines the impact of violating the equal-effect assumption, namely the assumption in (6) that the covariate effect θ_0 is shared by the source and target populations. To assess this sensitivity, we repeated the simulation experiments under a logistic link with an added effect shift δ, so that the risk model in (6) becomes

$$\mathbb{P}\left(Y = 1|X, S\right) = \varphi\left(\Delta_0(1 - S) + (1 + \delta S){\theta_0}^T X\right),$$

for $\delta = 0, 0.1, 0.3, 0.5$.

This violation affects the proposed method through two channels. First, the posterior risk $\mathbb{P}\left(Y = 1|X, S\right)$ is misspecified, since the fitted model assumes a common covariate effect across the two populations. Second, because the sample weights used to adjust for the $Y|X$ shift depend on this posterior risk, the estimated weights are also biased. To separate these two effects, we compare the proposed procedure with a reference procedure that uses the same misspecified posterior risk but replaces the estimated sample weights with the true weights. Therefore, the comparison with this reference isolates the additional loss due to biased weighting, rather than the total loss from equal-effect violation.

As shown in Figure S9.6a, the proposed procedure has larger TPR control error than the true-weight reference across all three covariate-shift settings, indicating that biased weighting further degrades TPR control beyond the posterior misspecification already shared by both procedures. Likewise, Figure S9.6b summarizes the additional loss in FPR due to biased weighting, measured as the achieved FPR under the proposed procedure minus that under the reference procedure using the same misspecified posterior risk but the true sample weights. Positive values therefore indicate extra FPR incurred by weight misspecification alone.

Overall, the effect shift does not appear to substantially degrade the overall TPR–FPR performance (Figure S9.7). Even under this violation, the proposed method remains more effective than the competing alternatives, although its advantage becomes less stable as the effect shift increases.

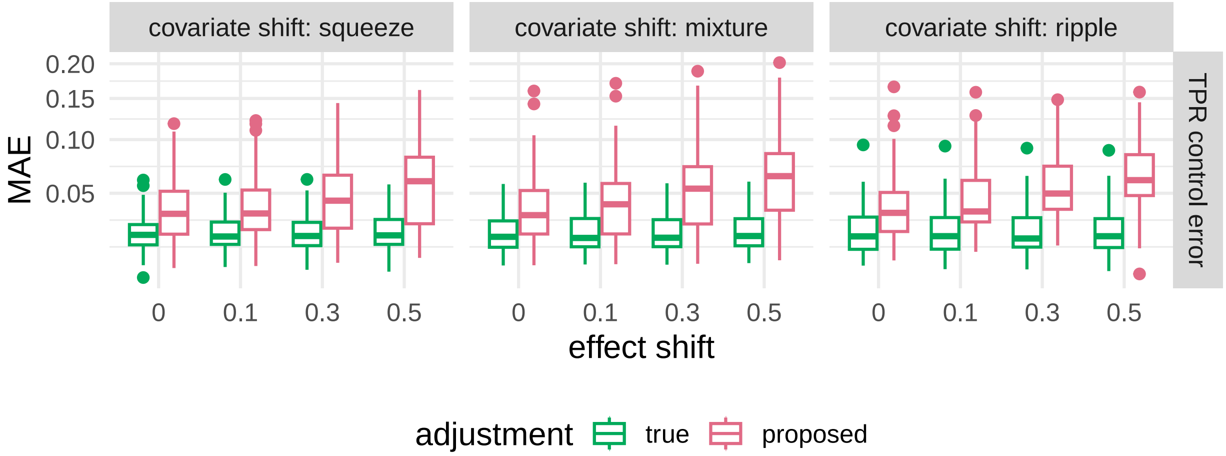

(a) MAE in retaining the requested TPR levels.

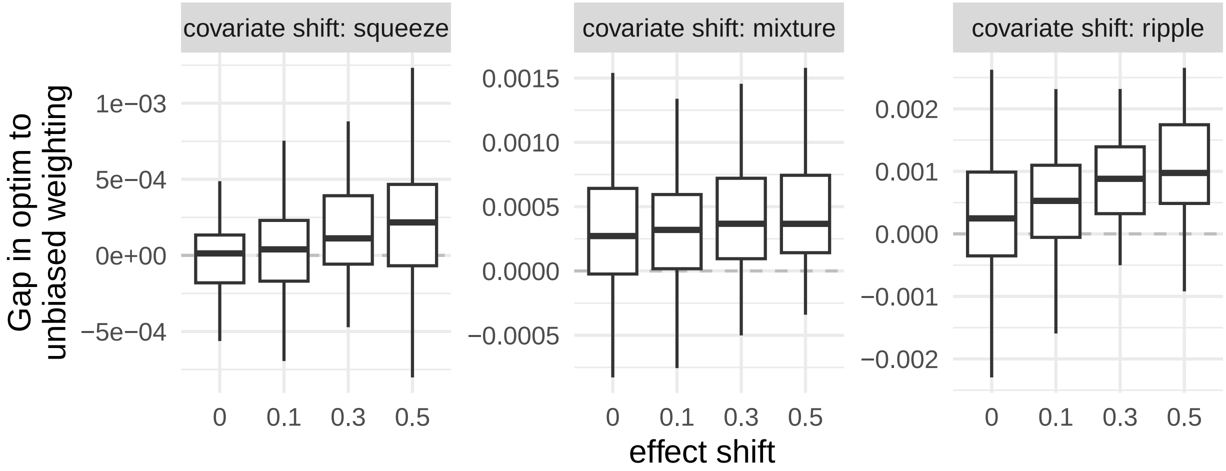

(b) Additional FPR loss due to biased weighting, defined as the achieved FPR under the proposed procedure minus that under a reference procedure using the same misspecified posterior risk but the true sample weights. Outliers are hidden for illustration.

Figure S9.6: Performance impact of equal-effect assumption violation to different extent. Each dot represents one repeat; figures show 100 repeated experiments.

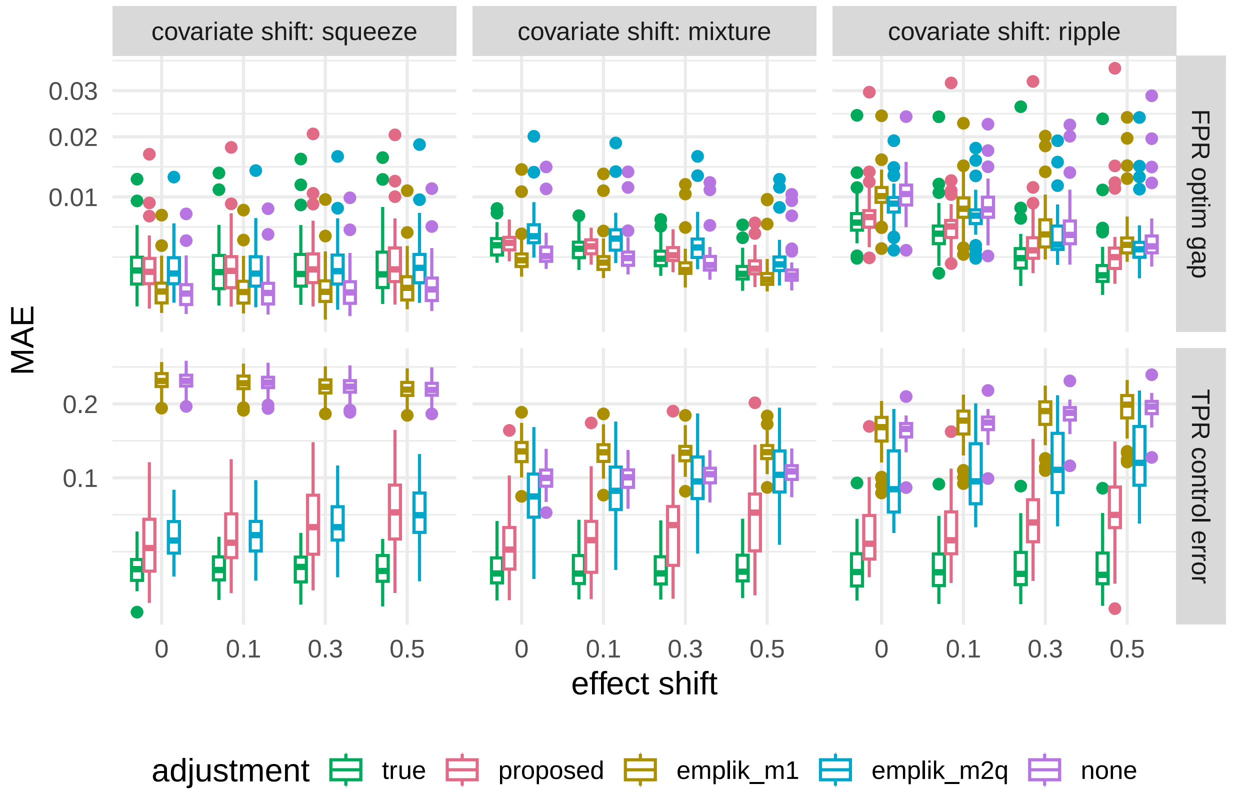

Figure S9.7: In analogy to Figure 1 under a logistic link, performance impact of equal-effect assumption violation to different extent. Each dot represents one repeat; figures show 100 repeated experiments.

S10 PCA3 Data

We used 200 repeats of 5-fold cross-validation to assess the performance of the proposed shift-adjustment method. Each one of the 5 folds served as a held-out fold iteratively. For each iteration, we estimated the rule from the 4 non-held-out folds and assessed its performance on a testing set drawn from the held-out fold. The testing set only includes observations from the target racial group. The shift-adjustment process reuses observations from the target population in the source sample (the 4 non-held-out folds), so no information from the actual testing sample was leaked. In the end, we pool across all 5 folds in one repeat by averaging true or false positive rates.

Similar to the simulation, the empirical likelihood was constructed by aligning the average and quantiles of the covariates among the source and target populations. $g(X_i^{\mathrm{sr}}, \theta) = X_i^{\mathrm{sr}} - \mathbb{E}X^{\mathrm{tg}} \in \mathbb{R}^4$.

The following Figure S10.1 supplements the Figure 3 in the main text. The MAE in Figure 4 essentially averages over the x-axis of Figure S10.1.

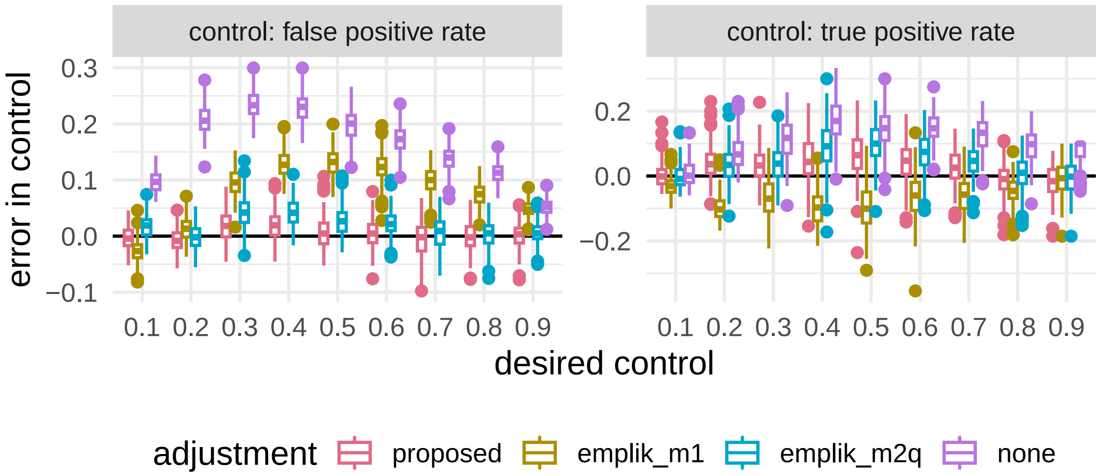

Figure S10.1: Errors (achieved minus expected) in desired control for the Black population.

We next consider the stability of estimated models. Figure S10.2 and Figure S10.3a suggest that the estimated risk model components (6) are relatively stable, which is consistent with our assumption that the conditional risk $\mathbb{P}(Y = 1|X)$ remains identical except a potential shift in the baseline risk (Δ_0). Figure S10.3b further suggests such a baseline shift is insignificant (by and large $\Delta_0 = 0$). Note that the covariates from Table 1 were first scaled before fitting the single index model.

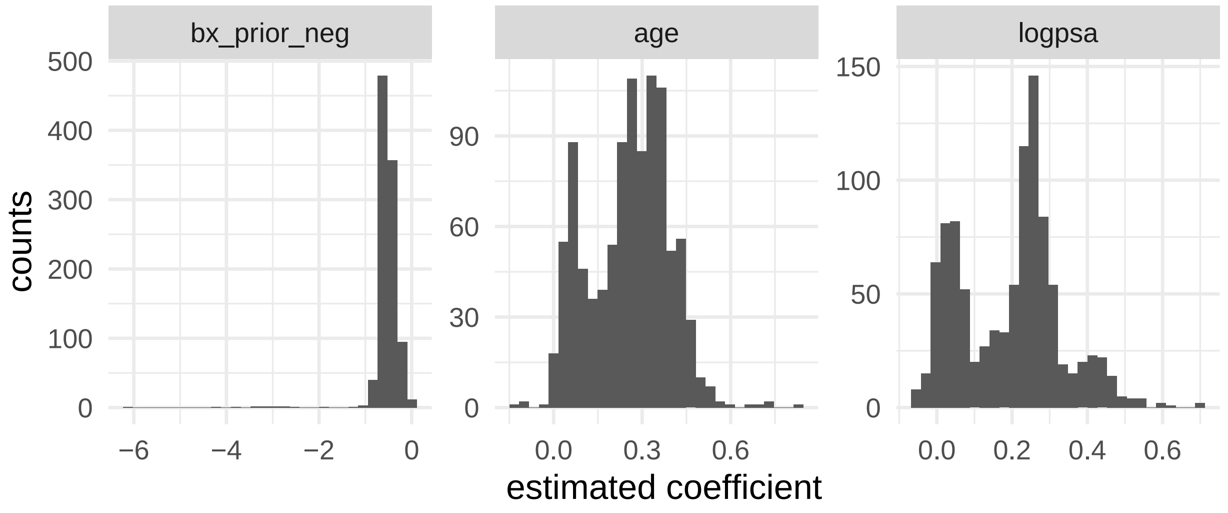

Figure S10.2: Histogram of estimated coefficients $\hat{\theta}$ in the single index model (6) while assessing racial disparity of the PCA3 data over repeated 5-fold cross-validation. The coefficient for the RNA marker is omitted since it is set to 1 for identifiability.

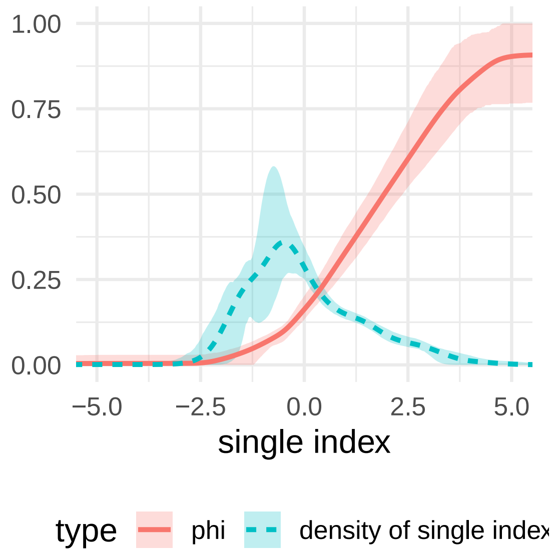

(a) Mean and pointwise 95% band of the estimated φ (red) and density of the single index for (6) while assessing racial disparity of the PCA3 data.

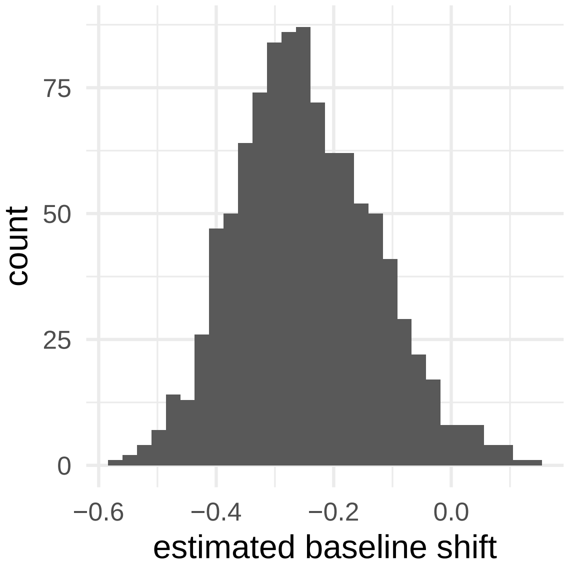

(b) Histogram for the estimated baseline shift $\hat{\Delta}_0$ while adjusting for the Black population.

Figure S10.3: Variability of estimated risk model components over repeated 5-fold cross-validation.

References

Boyd, S. P. and L. Vandenberghe (2004). *Convex Optimization.* Cambridge, UK ; New York: Cambridge University Press.

Casella, G. and R. L. Berger (2002). *Statistical Inference* (2nd ed ed.). Australia ; Pacific Grove, CA: Thomson Learning.

Chiou, J.-M. and H.-G. Müller (1998, December). Quasi-likelihood regression with unknown link and variance functions. *Journal of the American Statistical Association 93*(444), 1376–1387.

Chu, J., W. Lu, and S. Yang (2022, January). Targeted optimal treatment regime learning using summary statistics.

Delecroix, M., M. Hristache, and V. Patilea (2000). Optimal smoothing in semiparametric index approximation of regression functions. Working Paper 2000,4, SFB 373 Discussion Paper.

Delecroix, M., M. Hristache, and V. Patilea (2006, March). On semiparametric M-estimation in single-index regression. *Journal of Statistical Planning and Inference 136*(3), 730–769.

Devroye, L., L. Györfi, and G. Lugosi (1996). *A Probabilistic Theory of Pattern Recognition.* New York: Springer.

Giné, E. and A. Guillou (2002). Rates of strong uniform consistency for multivariate kernel density estimators. *Annales de l'Institut Henri Poincaré 38*, 907–921.

Han, P. and J. F. Lawless (2019). Empirical Likelihood Estimation Using Auxiliary Summary Information with Different Covariate Distributions. *Statistica Sinica*.

Hansen, B. E. (2008, June). Uniform convergence rates for kernel estimation with dependent data. *Econometric Theory 24*(3), 726–748.

Ichimura, H. (1993, July). Semiparametric least squares (SLS) and weighted SLS estimation of single-index models. *Journal of Econometrics 58*(1), 71–120.

Kanamori, T., S. Hido, and M. Sugiyama (2009). A Least-squares Approach to Direct Importance Estimation. *Journal of Machine Learning Research 10*(48), 1391–1445.

Klein, R. W. and R. H. Spady (1993). An efficient semiparametric estimator for binary response models. *Econometrica 61*(2), 387–421.

Kosorok, M. R. (2008). *Introduction to Empirical Processes and Semiparametric Inference.* Springer Series in Statistics. New York, NY: Springer.

Li, Q. and J. S. Racine (2007). *Nonparametric Econometrics: Theory and Practice.* Princeton, N.J: Princeton University Press.

Neyman, J., E. S. Pearson, and K. Pearson (1933). On the problem of the most efficient tests of statistical hypotheses. *Philosophical Transactions of the Royal Society of London. Series A, Containing Papers of a Mathematical or Physical Character 231*(694-706), 289–337.

Owen, A. (1990, March). Empirical likelihood ratio confidence regions. *The Annals of Statistics 18*(1).

Sugiyama, M., S. Nakajima, H. Kashima, P. Buenau, and M. Kawanabe (2007). Direct Importance Estimation with Model Selection and Its Application to Covariate Shift Adaptation. In *Advances in Neural Information Processing Systems*, Volume 20. Curran Associates, Inc.

Tong, X. (2013). A plug-in approach to Neyman-Pearson classification. *Journal of Machine Learning Research 14*(92), 3011–3040.

Zheng, J., Y. Zheng, and L. Hsu (2021, March). Risk projection for time-to-event outcome leveraging summary statistics with source individual-level data. *Journal of the American Statistical Association 0*(0), 1–13.
